# Dehydroabietyl squaramide incorporating chiral pyrrolidine for highly diastereo- and enantioselective Michael reaction between cyclohexanone and β-nitrostyrenes

**DOI:** 10.1039/d5ra06081h

**Published:** 2025-10-01

**Authors:** Zhen-Wei Zhang, Kai Xiong, Shao-Wu Liu, Yan-Qiu Deng

**Affiliations:** a College of Pharmacy, Guangxi Innovation Center of Zhuang Yao Medicine, Guangxi University of Chinese Medicine Nanning 530200 China dengyanqiu0501@163.com; b Guangxi University Engineering Research Center of Characteristic Traditional Chinese Medicine and Ethnic Medicine Nanning 530200 China

## Abstract

Through an efficient two-step synthetic strategy, we synthesized two novel dehydroabietyl pyrrolidin-2-yl squaramides, which were evaluated for their ability to catalyze the asymmetric Michael addition of cyclohexanone to β-nitrostyrenes. The (*R*)-pyrrolidin-2-yl substituted dehydroabietyl squaramide emerged as the superior catalyst, facilitating the asymmetric synthesis of the corresponding adducts with high yields (87–98%) and good to excellent stereoselectivity (up to >99 : 1 *syn*/*anti* ratio, 99% ee).

## Abstract 2

The (*R*)-pyrrolidin-2-yl-substituted dehydroabietyl squaramide served as an efficient organocatalyst for the asymmetric Michael addition of cyclohexanone to β-nitrostyrenes with high yields and good to excellent stereoselectivity.

## Graphical abstract

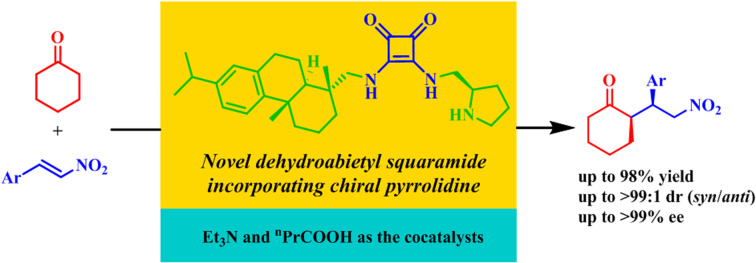

## Introduction

**Fig. 1:**
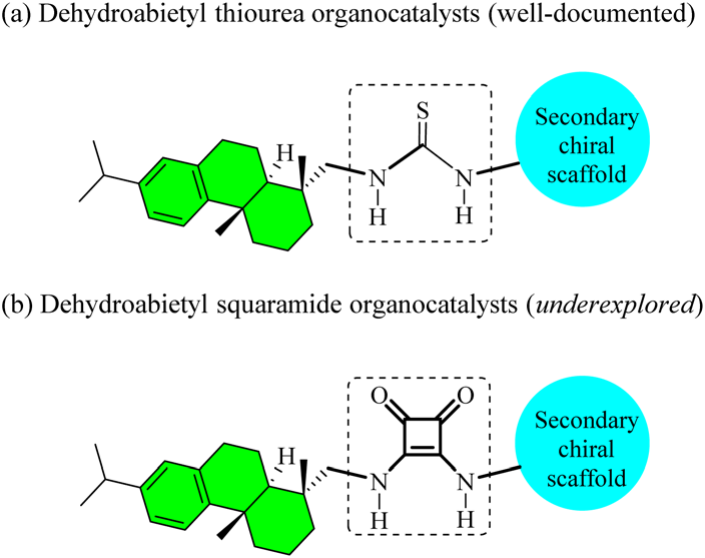
Bifunctional dehydroabietyl thioureas and squaramides.

The advent of asymmetric organocatalysis has revolutionized the construction of optically pure bioactive molecules and natural products,^1,2^ stimulating much more efforts toward catalyst innovation. Among the privileged scaffolds, chiral bifunctional dehydroabietyl thioureas (Fig. 1a) and squaramides (Fig. 1b) have demonstrated remarkable efficiency in diverse enantioselective transformations.^3^ These structurally cognate catalysts share a common dehydroabietyl framework integrated with a secondary chiral element, enabling stereocontrol *via* double hydrogen-bonding interactions. While dehydroabietyl thioureas have been thoroughly investigated, their squaramide counterparts have received comparatively less attention.

**Fig. 2:**
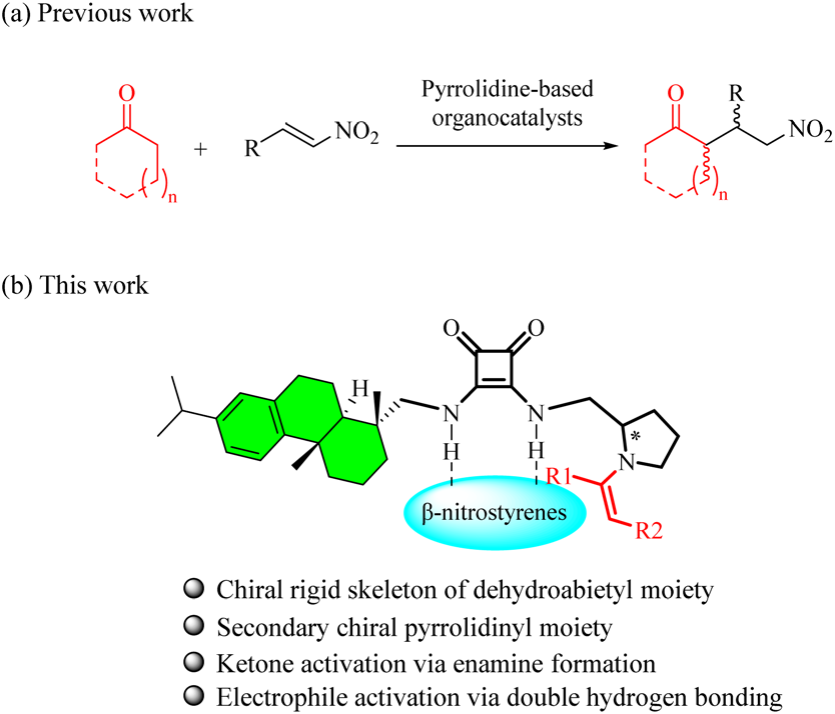
Design of dehydroabietyl pyrrolidin-2-yl squaramides.

Since List's groundbreaking discovery of proline-catalyzed direct enantioselective Michael reactions between unactivated ketones and nitroolefins,^4^ diverse pyrrolidine-based catalysts have been developed for various organocatalytic asymmetric conjugate additions.^5–18^ Among these, pyrrolidinyl (thio)ureas and squaramides have emerged as privileged scaffolds for promoting the asymmetric Michael reaction of cyclic ketones with nitroolefins (Fig. 2a). Nevertheless, systematic exploration of pyrrolidinyl squaramide architectures is still limited.

Capitalizing on these advancements and our continuous investigation of dehydroabietyl-based squaramide catalysts,^3^ we envisioned that strategic unification of chiral dehydroabietyl and pyrrolidin-2-yl structural elements *via* a squaramide tether might engender novel bifunctional organocatalysts (Fig. 2b). To the best of our knowledge, this particular molecular architecture, represents uncharted territory in organocatalyst design. Herein, we disclose the catalytic efficacy of chiral pyrrolidine-embedded dehydroabietyl squaramides in the asymmetric conjugate addition of cyclohexanone to β-nitrostyrenes.

## Results and discussion

### Synthesis of the target dehydroabietyl squaramides

**Scheme 1:**
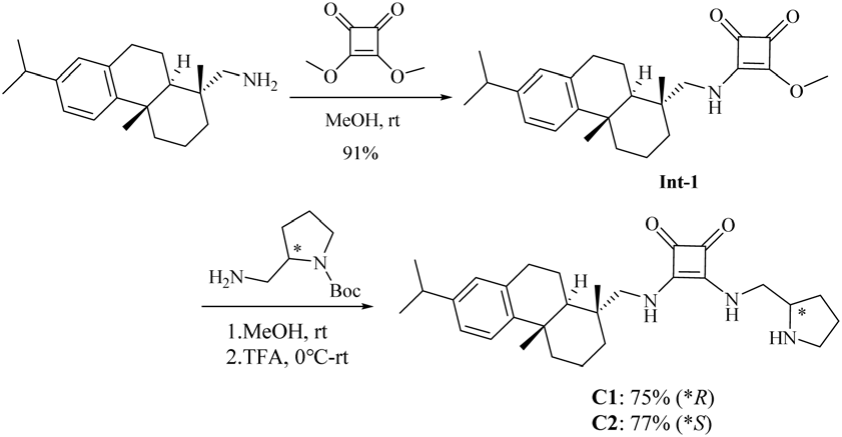
Synthetic route for squaramides C1 and C2.

Squaramides C1 and C2 were readily synthesized from commercially available dehydroabietylamine and (*R*)/(*S*)-1-Boc-2-(aminomethyl)pyrrolidine in two steps (Scheme 1).

### Asymmetric conjugate addition

We initially evaluated squaramides C1 and C2 in the model reaction of cyclohexanone 1 and nitroolefin 2a (Table 1).

**Table 1:** Optimization of reaction conditions^a^

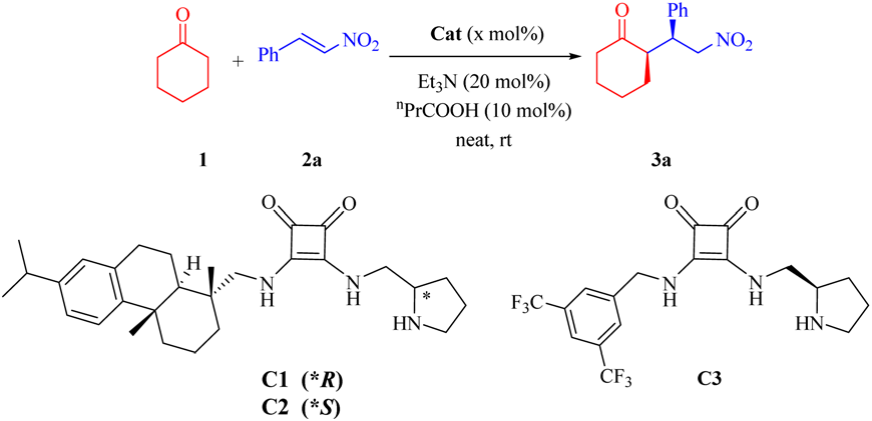						
Entry	Cat	*x*	Time (h)	Yield^b^ (%)	Dr^c^ (*syn*/*anti*)	Ee^d^ (%)
1	C1	10	20	98	97 : 3	91
2	C2	10	20	98	94 : 6	−78
3	C3	10	20	94	96 : 4	91
4^e^	C1	10	48	—	—	—
5^f^	C1	10	48	—	—	—
6^g^	—	—	48	—	—	—
7^h^	—	—	48	—	—	—
8^i^	C1	10	72	95	90 : 10	83
9^j^	C1	10	72	—	—	—
10^k^	C1	10	72	—	—	—
11^l^	C1	10	72	95	90 : 10	85
12^m^	C1	10	48	98	>99 : 1	97
13^m^	C3	10	144	90	>99 : 1	94
14^m^	C1	5	96	97	>99 : 1	98

a All reactions were performed with 1 (20 eq.), 2a (0.2 mmol), cat (*x* mol%), Et_3_N (20 mol%) and ^*n*^PrCOOH (10 mol%), neat, r.t.

b Isolated yields.

c Determined by HPLC analysis.

d ee of *syn* diastereomer, determined by HPLC analysis.

e Without ^*n*^PrCOOH.

f Without Et_3_N and ^*n*^PrCOOH.

g Only Et_3_N was used.

h Only Et_3_N and ^*n*^PrCOOH were used.

i CF_3_COOH (10 mol%).

j PhCOOH (10 mol%).

k MeOH.

l DCM.

m 0 °C.

As shown in Table 1, squaramides C1–C3 effectively catalyzed the reaction under standard conditions (catalyst, Et_3_N, ^*n*^PrCOOH, neat, r.t.),^11^ delivering the *syn*-adducts in high yields, with excellent diastereoselectivities and good to excellent enantioselectivities (entries 1–3). The stereochemistry of the dehydroabietyl moiety in the new squaramides demonstrated better compatibility with the (*R*)-pyrrolidin-2-yl moiety (entry 1 *vs.* 2) and a slight advantage compared to catalyst C3 bearing the 3,5-bis(trifluoromethyl)phenyl group (entry 1 *vs.* 3). Control experiments revealed that C1, bases, and acids are essential for the reaction, as no conversion occurred in their absence (entries 4–7). Subsequent screening of alternative Brønsted acids and other solvents failed to improve upon the standard conditions (entries 8–11). Lowering the reaction temperature significantly enhanced stereocontrol (entries 12 and 13), underscoring the key role of the dehydroabietyl framework in catalytic activity. Furthermore, reducing the catalyst loading to 5 mol% necessitated a prolonged reaction time, while high catalytic efficiency was still effectively maintained (entry 14).

### Substrate scope

The optimized reaction conditions were applied to a range of β-nitrostyrenes (Table 2).

**Table 2:** Scope of the Michael addition by using C1^a^

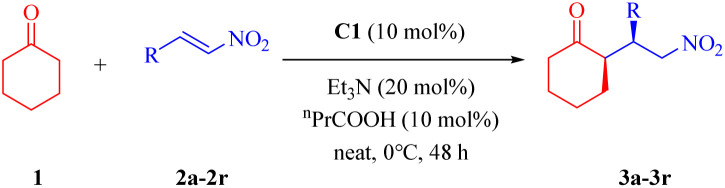				
Entry	R	Yield^b^ (%)	Dr^c^ (*syn*/*anti*)	Ee^d^ (%)
1	C_6_H_5_·3a	98	>99 : 1	97
2	4-OCH_3_C_6_H_4_·3b	95	95 : 5	92
3	4-CH_3_C_6_H_4_·3c	93	>99 : 1	94
4	4-CNC_6_H_4_·3d	92	87 : 13	96
5	4-FC_6_H_4_·3e	96	>99 : 1	94
6	4-ClC_6_H_4_·3f	95	>99 : 1	96
7	4-BrC_6_H_4_·3g	93	87 : 13	92
8	3-OCH_3_C_6_H_4_·3h	92	92 : 8	94
9	3-FC_6_H_4_·3i	93	>99 : 1	95
10	3-BrC_6_H_4_·3j	95	>99 : 1	94
11	2-OCH_3_C_6_H_4_·3k	93	>99 : 1	>99
12	2-FC_6_H_4_·3l	90	87 13	93
13	2-ClC_6_H_4_·3m	95	80 : 20	>99
14	2-BrC_6_H_4_·3n	94	>99 : 1	97
15	2,4-diClC_6_H_3_·3o	95	>99 1	98
16	2-Naphthyl·3p	92	>99 : 1	92
17	2-Thienyl·3q	93	>99 : 1	92
18	2-Furyl·3r	87	87 : 13	78

a All reactions were performed with 1 (20 eq.), 2a–2r (0.2 mmol), C1 (10 mol%), Et_3_N (20 mol%) and ^*n*^PrCOOH (10 mol%), neat, 0 °C.

b Isolated yields.

c Determined by HPLC analysis.

d ee of *syn* diastereomer, determined by HPLC analysis.

Generally, β-nitrostyrenes with 4-, 3-, or 2-substituted phenyl groups (electron-donating/withdrawing groups) delivered high yields (90–98%), good to excellent diastereo- (up to >99 : 1 *syn*/*anti*) and excellent enantioselectivities (92–>99% ee). Substrates bearing 2-naphthyl and heterocyclic moieties (2-thienyl and 2-furyl) also showed favorable reactivity, although the 2-furyl derivatives exhibited diminished selectivity (87 : 13 dr, 78% ee). The observed decrease in stereoselectivity for the 2-furyl substrate may be attributed to the stronger tendency of oxygen atom, relative to sulfur atom in the 2-thienyl substrate, to participate in hydrogen bonding.^19^ The oxygen atom in the furyl ring might form additional hydrogen-bonding interactions within the catalytic environment, which could interfere with the catalyst's ability to exert stereochemical control, consequently leading to lower stereoselectivity. Moreover, the stereochemical assignments of the Michael adducts were established through comparative analysis of their NMR spectroscopic data and specific optical rotations with those of structurally characterized analogues documented in prior studies (see SI).^11*a*,13,14,16^

**Scheme 2:**
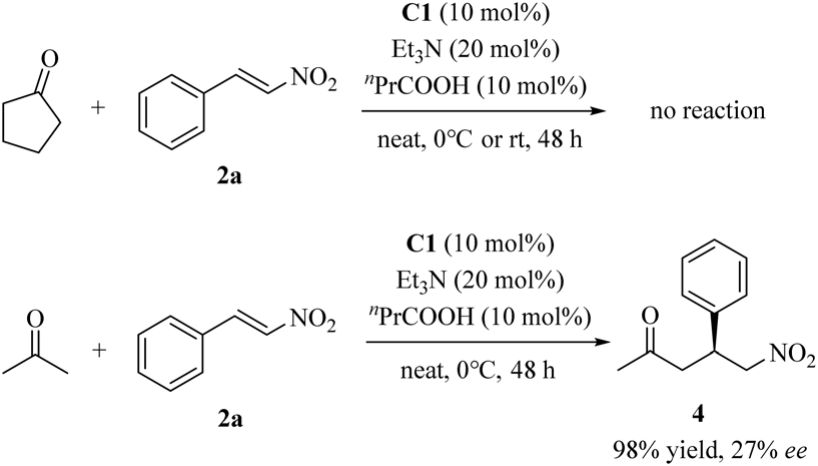
Conjugate additions of other ketones to 2a.

The asymmetric additions of other ketones to β-nitrostyrene 2a were also examined under the optimized reaction conditions. As illustrated in Scheme 2, cyclopentanone exhibited extremely poor reactivity and did not yield the desired adduct even at room temperature. Acetone afforded the desired product in an excellent yield (98%), yet with low enantioselectivity (27% ee). These findings may be rationalized by considering the distinct energy landscapes associated with the nucleophilic addition of the pyrrolidine moiety to the carbonyl group of structurally diverse ketones, which governs the kinetics of enamine generation,^11*a*^ as well as the differential steric hindrance encountered during the C–C bond-forming event between the *in situ*-generated enamine and the nitroolefin acceptor.

### Scale-up synthesis

**Scheme 3:**
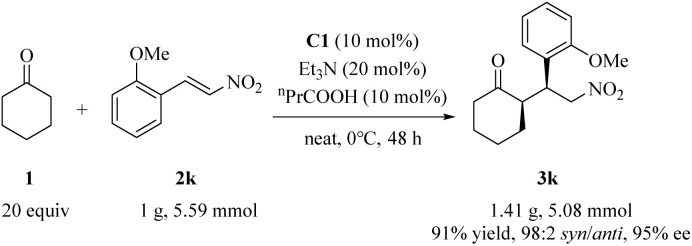
Gram-scale preparation of the adduct 3k.

A gram-scale reaction of cyclohexanone 1 with substrate 2k using 10 mol% of catalyst C1 afforded the adduct 3k in 91% yield with excellent stereoselectivities (98 : 2 *syn*/*anti* and 95% ee), albeit with a slight decrease in selectivity (Scheme 3).

### Proposed catalytic mechanism

**Scheme 4:**
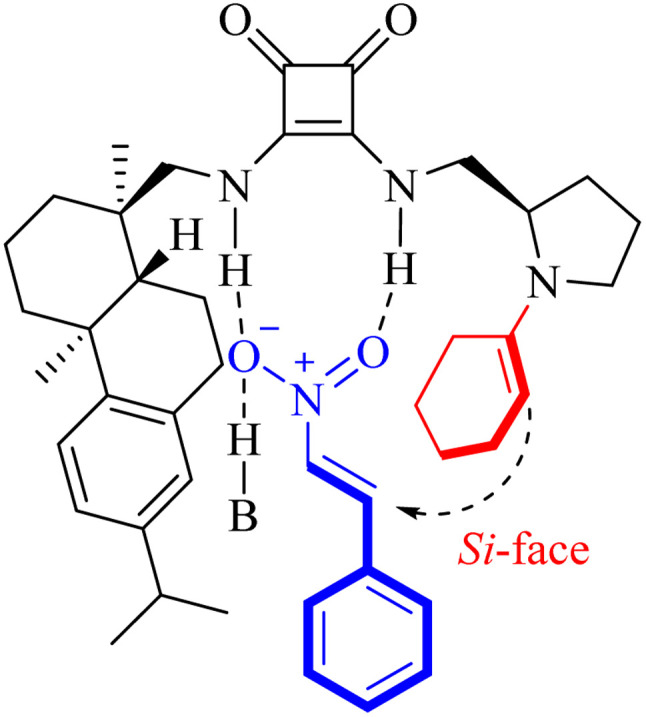
Possible transition-state model.

As outlined in Scheme 4, the proposed catalytic mechanism may proceed *via* a cooperative bifunctional activation process facilitated by the Et_3_N/^*n*^PrCOOH system, according to our experimental data and literature precedents.^11–16^ While further studies are needed to fully understand the details, this steric control model offers a plausible explanation for the high level of stereoselectivity observed in the reaction. The pathway may follow a sequence in which: (i) the pyrrolidine moiety generates a catalytically active enamine intermediate with the carbonyl substrate; (ii) concurrently, the squaramide moiety organizes the nitroolefin electrophile and the base *via* well-defined H-bonding interactions; while (iii) the sterically demanding dehydroabietyl scaffold confers facial selectivity, directing the enamine attack preferentially toward the *Si*-face.

## Conclusions

In summary, we have developed a new highly efficient chiral bifunctional organocatalyst, featuring a dehydroabietyl (*R*)-pyrrolidin-2-yl squaramide architecture that is strategically integrated to enhance catalytic performance. The catalyst demonstrated remarkable performance in the asymmetric Michael addition of cyclohexanone to diverse β-nitrostyrenes, enabling the synthesis of valuable chiral γ-nitroketone derivatives with high efficiency (up to 98% yield) and good to excellent stereocontrol (up to >99 : 1 *syn*/*anti* and >99% ee). The versatility of this catalytic system in other asymmetric transformations, along with detailed mechanistic studies (*e.g.*, computational modeling) is currently under investigation in our ongoing research.

## Experimental section

### General information

All reactions were conducted directly under ambient air unless otherwise stated. All reagents were obtained from commercial sources and used without further purification. The isolation and purification of all reaction products were carried out by column chromatography on silica gel (200–300 mesh). The reaction progress was monitored by thin-layer chromatography (TLC) using precoated silica gel 60 GF254 plates and visualized under UV light. NMR spectra were recorded by using Bruker Advance III spectrometer (500 MHz) in CDCl_3_ and CF_3_COOD. All enantiomeric excesses were determined by HPLC analysis using chiral stationary phases IA-H or AD-H in a Shimadzu LC-20AT chromatogram with hexane/2-propanol as eluent. High-resolution mass spectra were recorded using a SCIEX ESI-QTOF. The optical rotation measurements were conducted using an IP-digi 300/8 digital polarimeter, and the melting points were determined with a WRS-3 melting-point apparatus. Catalyst C3 was synthesized according to the reported synthetic procedure.^12^

### Preperation of dehydroabietyl squaramides C1–C2

To a solution of dehydroabietylamine (2.85 g, 10 mmol) in methanol was added dimethyl squarate (1.56 g, 11 mmol), and the mixture was stirred at room temperature for 48 h. The resulting precipitate was triturated with methanol, filtered under reduced pressure and washed with methanol to afford a light pink solid, Int-1 (3.72 g, 91% yield).

To a stirred solution of (*R*)- or (*S*)-*tert*-butyl-2-(aminomethyl) pyrrolidine-1-carboxylate (0.1 g, 0.5 mmol) in methanol was added the intermediate Int-1 (0.22 g, 0.55 mmol) at room temperature. After 48 h, the white precipitate was filtered and dried. The collected solid was subsequently dissolved in dichloromethane, and trifluoroacetic acid (10 equiv.) was added dropwise at 0 °C. The resulting solution was stirred at room temperature for 48 h. The pH was adjusted using aqueous Na_2_CO_3_ solution. The mixture was extracted with dichloromethane (3 × 20 mL) and washed by brine. The combined extracts were dried over anhydrous Na_2_SO_4_, and purified through column chromatography on silica gel (dichloromethane/methanol = 100 : 1–10 : 1) to give the desired catalysts.

#### 3-((((1*R*, 4a*S*, 10a*R*)-7-isopropyl-1,4a-dimethyl-1,2,3,4,4a,9,10,10a-octahydrophenanthren-1-yl)methyl)amino)-4-((((*R*)-pyrrolidin-2-yl)methyl)amino)cyclobut-3-ene-1,2-dione (C1)

White solid, (0.152 g, 0.32 mmol, 75% yield). m.p. 203.5–204.2 °C. [*α*]_D_^20^ = −19.1 (c 0.5, 2% TFA in CH_2_Cl_2_). ^1^H NMR (500 MHz, CDCl_3_/CF_3_COOD) *δ* 7.17 (d, *J* = 8.2 Hz, 1H), 7.02 (dd, *J* = 8.2, 2.0 Hz, 1H), 6.90 (d, *J* = 2.0 Hz, 1H), 4.09–4.00 (m, 3H), 3.62–3.52 (m, 3H), 3.53–3.42 (m, 1H), 2.99–2.88 (m, 1H), 2.87–2.74 (m, 2H), 2.37–2.30 (m, 2H), 2.30–2.09 (m, 3H), 1.94–1.65 (m, 6H), 1.53–1.25 (m, 5H), 1.23 (s, 6H), 1.21 (s, 3H), 0.99 (s, 3H). ^13^C NMR (126 MHz, CDCl_3_) *δ* 178.75, 178.44, 169.25, 166.01, 146.92, 146.26, 134.56, 127.13, 124.29, 61.79, 61.73, 57.01, 56.86, 47.23, 47.14, 47.06, 45.73, 38.12, 37.65, 35.61, 33.61, 29.88, 27.69, 25.30, 23.98, 23.49, 23.46, 18.92, 18.30, 17.74. HR-MS-EI (*m*/*z*): calcd for C_29_H_41_N_3_O_2_ [M–H]^−^: 462.3126; found: 462.3122.

#### 3-((((1*R*,4a*S*,10a*R*)-7-isopropyl-1,4a-dimethyl-1,2,3,4,4a,9,10,10a-octahydrophenanthren-1-yl)methyl)amino)-4-((((*S*)-pyrrolidin-2-yl)methyl)amino)cyclobut-3-ene-1,2-dione (C2)

White solid, (0.156 g, 0.34 mmol, 77% yield). m.p. 173.8–174.0 °C. [*α*]_*n*_^20^ = +16.0 (c 0.5, 1% TFA in CH_2_Cl_2_). ^1^H NMR (500 MHz, CDCl_3_/CF_3_COOD) *δ* 7.18 (d, *J* = 8.1 Hz, 1H), 7.03 (dd, *J* = 8.2, 2.1 Hz, 1H), 6.91 (d, *J* = 2.1 Hz, 1H), 4.17–4.01 (m, 3H), 3.68 (d, *J* = 13.5 Hz, 1H), 3.61–3.53 (m, 2H), 3.51–3.46 (m, 1H), 2.95 (dd, *J* = 17.2, 5.8 Hz, 1H), 2.90–2.75 (m, 2H), 2.42–2.30 (m, 2H), 2.30–2.03 (m, 3H), 1.90–1.68 (m, 6H), 1.55–1.26 (m, 5H), 1.23 (d, *J* = 5.3 Hz, 6H), 1.21 (s, 3H), 1.00 (s, 3H). ^13^C NMR (126 MHz, CDCl_3_) *δ* 178.16, 178.05, 169.32, 165.75, 146.97, 146.53, 134.63, 127.24, 124.42, 62.01, 57.29, 47.36, 46.08, 45.47, 38.26, 38.21, 37.77, 35.70, 33.73, 29.91, 27.74, 25.25, 23.92, 23.90, 23.49, 19.06, 18.29, 17.58. HR-MS-EI (*m*/*z*): calcd for C_29_H_41_N_3_O_2_ [M–H]^−^: 462.3126; found: 462.3124.

### General procedure for the catalytic asymmetric Michael addition

To a stirred solution of cyclohexanone (20 equiv.) were added triethylamine (20 mol%) and the catalyst (10 mol%) at room temperature. *n*-Butyric acid (10 mol%) was introduced after 30 min, and the mixture was further stirred for 15 min. Finally, β-nitroolefins (1 equiv.) were added at the designated temperature. Upon completion of the reaction, the mixture was purified by column chromatography using silica gel with a gradient elution of petroleum ether and ethyl acetate from 50 : 1 to 10 : 1, yielding the corresponding adducts.

## Author contributions

Conceptualization and funding acquisition, Z.-W. Zhang and Y.-Q. Deng; writing – original draft preparation, K. Xiong and S.-W. Liu; writing – review and editing, Z.-W. Zhang and Y.-Q. Deng.

## Conflicts of interest

The authors declare no competing financial interest.

## Supplementary Material

RA-015-D5RA06081H-s001

## Data Availability

The data of NMR, HRMS and HPLC spectra of the synthesized compounds supporting this article have been included as part of the supplementary information (SI). Supplementary information: NMR and HRMS spectra of the new catalysts, and the characterization data of all adducts. See DOI: https://doi.org/10.1039/d5ra06081h.
