# Supplementary material for: Dehydroabietyl squaramide incorporating chiral pyrrolidine for highly diastereo- and enantioselective Michael reaction between cyclohexanone and β-nitrostyrenes
Source: RSC Adv. 2025 Oct 1;15(43):36485–9. doi: 10.1039/d5ra06081h (PMC12487773; doi:10.1039/d5ra06081h)
Supplement: RA-015-D5RA06081H-s001 [file RA-015-D5RA06081H-s001.pdf]

Supplementary Information (SI) for RSC Advances.
This journal is © The Royal Society of Chemistry 2025

Supplementary Information

Dehydroabietyl squaramide incorporating chiral pyrrolidine for highly diastereo- and enantioselective Michael reaction between cyclohexanone and β-nitrostyrenes

Zhen-Wei Zhang[ab], Kai Xiong[§a], Shao-Wu Liu[§a], and Yan-Qiu Deng*[a]

a. College of Pharmacy, Guangxi Innovation Center of Zhuang Yao Medicine, Guangxi University of Chinese Medicine, Nanning, 530200, China.

b. Guangxi University Engineering Research Center of Characteristic Traditional Chinese Medicine and Ethnic Medicine, Nanning, 530200, China.

* Corresponding author. E-mail: dengyanqiu0501@163.com (Y.-Q. Deng)

[§] These two authors contribute equally.

Table of Contents

1. NMR spectra and HRMS of catalysts **C1** and **C2** ········· S2
2. Characterization data of products **3a**–**3r** ········· S6
3. NMR spectra of products **3a**–**3r** ········· S10
4. HPLC traces of racemic and chiral products **3a**–**3r** and **4** ········· S20

1. NMR spectra and HRMS of catalysts **C1** and **C2**

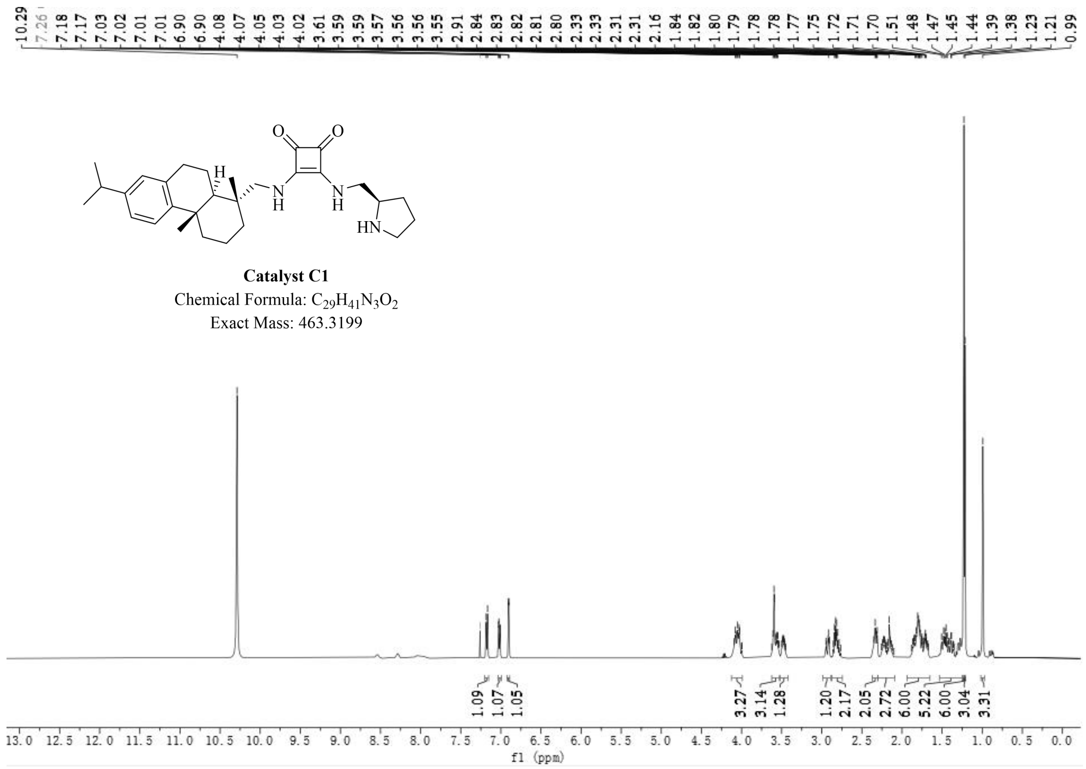

^{1}H NMR (500 MHz, $CDCl_3$: CF_3COOD = 4 : 1) spectrum of **C1**

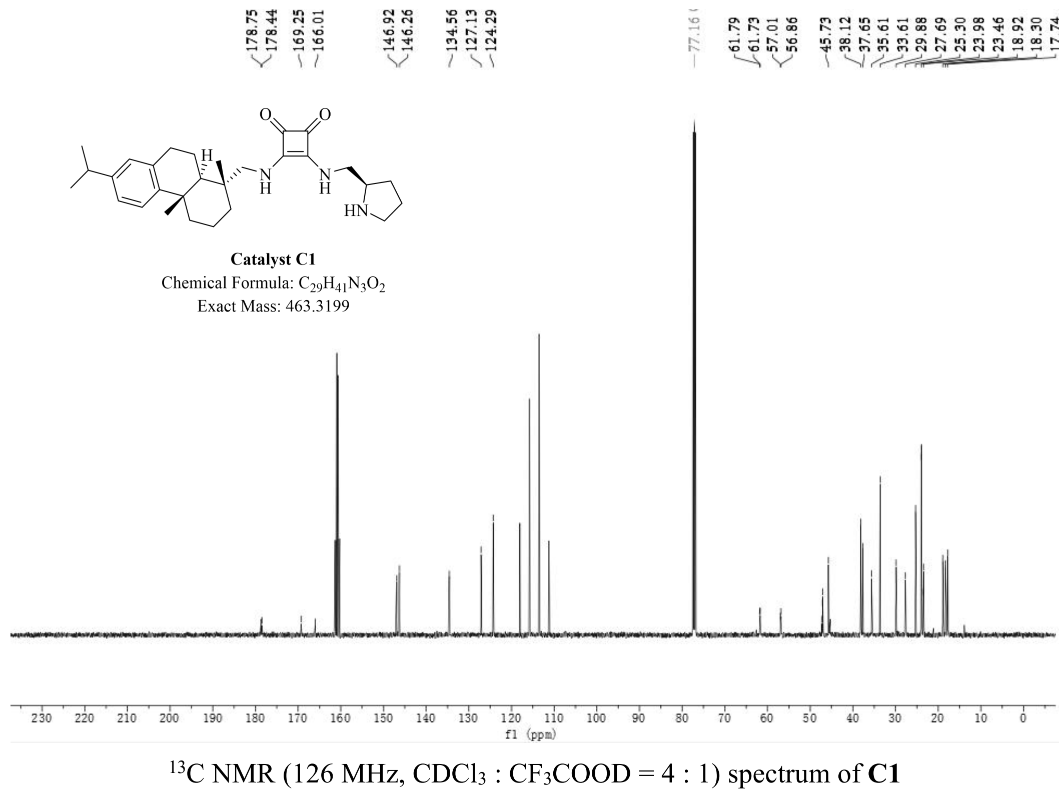

^{13}C NMR (126 MHz, $CDCl_3$: CF_3COOD = 4 : 1) spectrum of **C1**

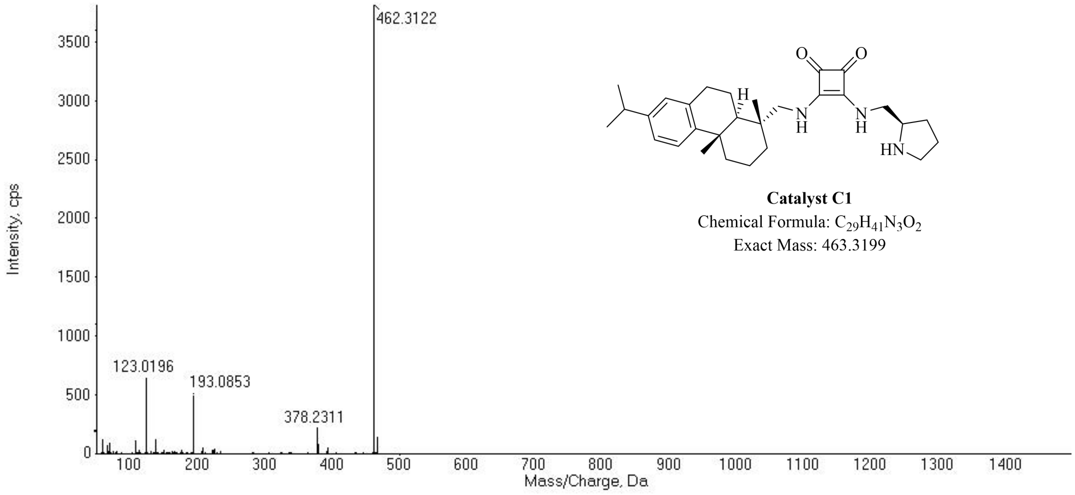
462.3122
123.0196
193.0853
378.2311
Intensity, cps
Mass/Charge, Da
Catalyst C1
Chemical Formula: C29H41N3O2
Exact Mass: 463.3199

HR-MS-EI of **C1**

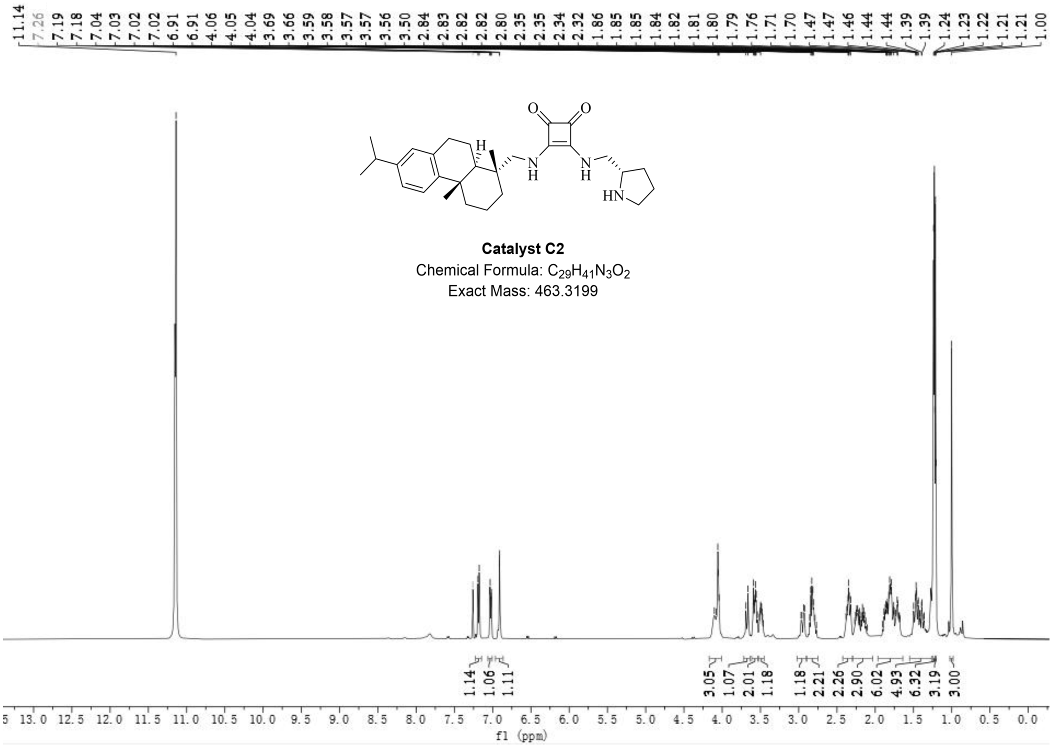

^{1}H NMR (500 MHz, $CDCl_3$: CF_3COOD = 4 : 1) spectrum of **C2**

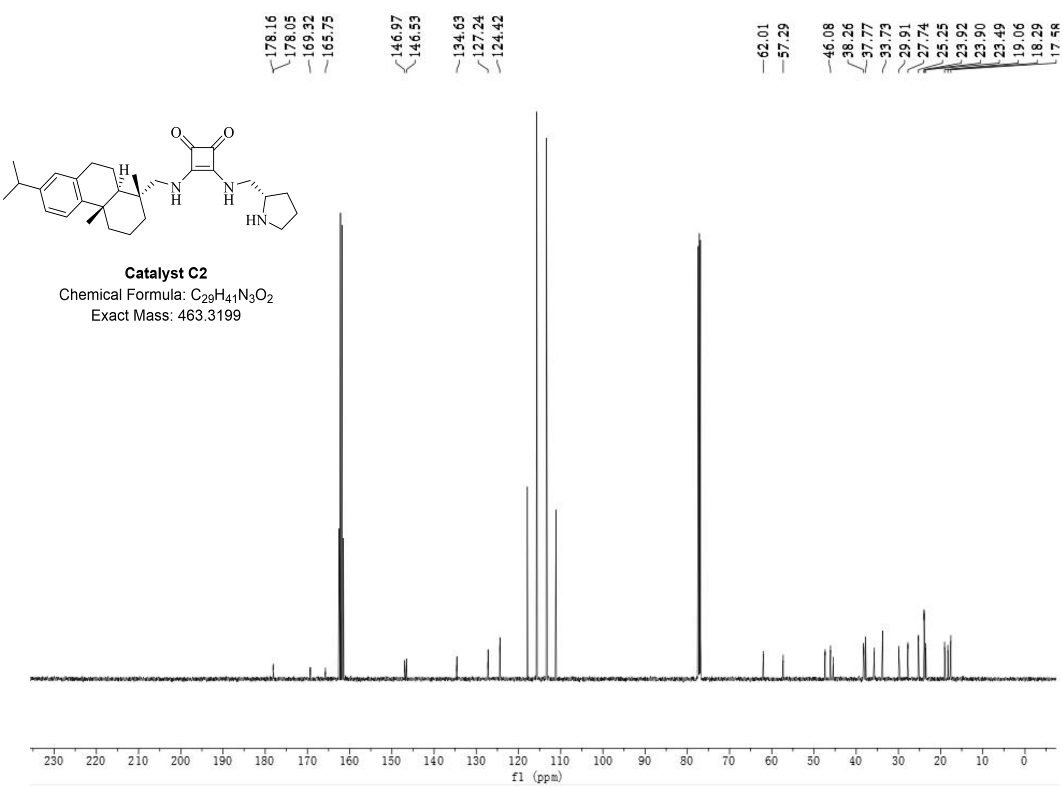

^{13}C NMR (126 MHz, $CDCl_3$: CF_3COOD = 4 : 1) spectrum of **C2**

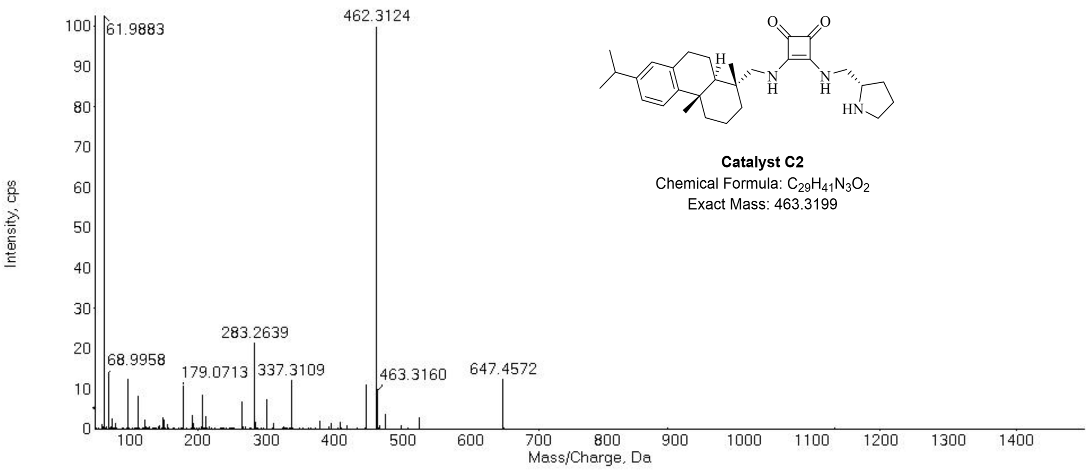
462.3124
61.9883
283.2639
68.9958
179.0713
337.3109
463.3160
647.4572
Intensity, cps
Mass/Charge, Da
Catalyst C2
Chemical Formula: C29H41N3O2
Exact Mass: 463.3199

HR-MS-EI of **C2**

2. Characterization data of products **3a**–**3r**

(*R*)-2-((*S*)-2-Nitro-1-phenylethyl)cyclohexan-1-one (3a)[1-8]

White solid, (0.048 g, 0.19 mmol, 98% yield), 97% *ee*, >99:1 *dr* (*syn*: *anti*). $[\alpha]_D^{20}$= +29.4 (c 0.2, $CHCl_3$). ^{1}H NMR (500 MHz, $CDCl_3$) δ 7.33 (dd, *J* = 8.1, 6.6 Hz, 2H), 7.28 (d, *J* = 8.2 Hz, 1H), 7.18 (dd, *J* = 7.1, 1.8 Hz, 2H), 4.94 (dd, J = 12.5, 4.5 Hz, 1H), 4.63 (dd, J = 12.5, 9.9 Hz, 1H), 3.79 – 3.73 (m, 1H), 2.72 – 2.65 (m, 1H), 2.50 – 2.50 (m, 1H), 2.42 –2.35 (m, 1H), 2.12 – 2.04 (m, 1H), 1.75 – 1.52 (m, 4H),1.19 – 1.24(m, 1H). HPLC analysis (chiralpak AD-H column, hexane:2-propanol = 90:10, flow rate = 1 mL/min, wavelength = 220 nm), t (major) = 9.98 min, t (minor) = 12.22 min.

(*R*)-2-((*S*)-1-(4-Methoxyphenyl)-2-nitroethyl)cyclohexan-1-one (3b)[2-4,6-8]

White solid, (0.050 g, 0.19 mmol, 95% yield), 92% *ee*, 95:5 *dr* (*syn*: *anti*). $[\alpha]_D^{20}$= +35.4 (c 0.31, $CHCl_3$). ^{1}H NMR (500 MHz, $CDCl_3$) δ 7.08 (d, *J* = 8.7 Hz, 2H), 6.84 (d, *J* = 8.7 Hz, 2H), 4.91 (dd, *J* = 12.3, 4.6 Hz, 1H), 4.58 (dd, *J* = 12.3, 10.0 Hz, 1H), 3.78 (s, 3H), 3.71 (td, *J* = 10.0, 4.6 Hz, 1H), 2.69 – 2.60 (m, 1H), 2.50 – 2.43 (m, 1H), 2.43 – 2.33 (m, 1H), 2.10 – 2.05 (m, 1H), 1.73 – 1.54 (m, 4H), 1.27 – 1.22 (m, 1H). HPLC analysis (chiralpak AD-H column, hexane:2-propanol = 95:5, flow rate = 1 mL/min, wavelength = 238 nm), t (major) = 21.05 min, t (minor) = 26.72 min.

(*R*)-2-((*S*)-2-Nitro-1-(p-tolyl)ethyl)cyclohexan-1-one (3c)[3-7]

White solid, (0.052 g, 0.19 mmol, 93% yield), 94% *ee*, >99:1 *dr* (*syn*: *anti*). $[\alpha]_D^{20}$= +17.6 (c 0.14, $CHCl_3$). ^{1}H NMR (500 MHz, $CDCl_3$) δ 7.12 (d, *J* = 7.8 Hz, 2H), 7.04 (d, *J* = 8.0 Hz, 2H), 4.92 (dd, *J* = 12.4, 4.6 Hz, 1H), 4.60 (dd, *J* = 12.4, 10.0 Hz, 1H), 3.71 (td, *J* = 9.9, 4.5 Hz, 1H), 2.72 – 2.62 (m, 1H), 2.50 – 2.44 (m, 1H), 2.42 – 2.34 (m, 1H), 2.31 (s, 3H), 2.10 – 2.04 (m, 1H), 1.75 – 1.55 (m, 4H), 1.28 – 1.23 (m, 1H).HPLC analysis (chiralpak AD-H column, hexane:2-propanol = 95:5, flow rate = 1 mL/min, wavelength = 254 nm), t (major) = 11.83 min, t (minor) = 15.30 min.

4-((*S*)-2-Nitro-1-((*R*)-2-oxocyclohexyl)ethyl)benzonitrile (3d)[8]

White solid, (0.050 g, 0.18 mmol, 92% yield), 96% *ee*, 87:13 *dr* (*syn*: *anti*). $[\alpha]_D^{20}$ = +26.8 (c 0.21, $CHCl_3$). ^{1}H NMR (500 MHz, $CDCl_3$) δ 8.21 (d, J = 8.6 Hz, 2H), 7.40 (d, J = 8.6 Hz, 2H), 5.00 (dd, J = 13.0, 4.4 Hz, 1H), 4.70 (dd, J = 13.0, 10.2 Hz, 1H), 3.94 (td, J = 9.8, 4.4 Hz, 1H), 2.76 – 2.68(m, 1H), 2.50 (d, J = 13.2 Hz, 1H), 2.42 – 2.36 (m, 1H), 2.17 – 2.09 (m, 1H), 1.85 – 1.79 (m, 1H), 1.71 – 1.55 (m, 3H), 1.33 – 1.22 (m, 1H). HPLC analysis (chiralpak AD-H column, hexane:2-propanol = 90:10, flow rate = 1 mL/min, wavelength = 220 nm), t (major) = 30.28 min, t (minor) = 48.82 min.

(*R*)-2-((*S*)-1-(4-Fluorophenyl)-2-nitroethyl)cyclohexan-1-one (3e)[3,7,8]

White solid, (0.051 g, 0.19 mmol, 96% yield), 94% *ee*, >99:1 *dr* (*syn: anti*). $[\alpha]_D^{20}$ = +21.3 (c 0.24, $CHCl_3$). ^{1}H NMR (500 MHz, $CDCl_3$) δ 7.14 (dd, *J* = 8.6, 5.4 Hz, 2H), 7.01 (t, *J* = 8.6 Hz, 2H), 4.93 (dd, *J* = 12.6, 4.5 Hz, 1H), 4.59 (dd, *J* = 12.5, 10.0 Hz, 1H), 3.76 (td, *J* = 9.9, 4.5 Hz, 1H), 2.68 – 2.62 (m, 1H), 2.49 – 2.45 (m, 1H), 2.41 – 2.34 (m, 1H), 2.11 – 2.06 (m, 1H), 1.73 – 1.57 (m, 4H), 1.28 – 1.22 (m, 1H). HPLC analysis (chiralpak AD-H column, hexane:2-propanol = 95:5, flow rate = 1 mL/min, wavelength = 254 nm), t (major) = 17.31 min, t_{minor} = 24.27 min.

(*R*)-2-((*S*)-1-(4-Chlorophenyl)-2-nitroethyl)cyclohexan-1-one (3f)[2-5,7,8]

White solid, (0.053 g, 0.19 mmol, 95% yield), 96% *ee*, >99:1 *dr* (*syn: anti*). $[\alpha]_D^{20}$= +37.2 (c 0.34, $CHCl_3$). ^{1}H NMR (500 MHz, $CDCl_3$) δ 7.30 (d, *J* = 8.4 Hz, 2H), 7.11 (d, *J* = 8.4 Hz, 2H), 4.93 (dd, *J* = 12.6, 4.5 Hz, 1H), 4.60 (dd, *J* = 12.6, 10.0 Hz, 1H), 3.75 (td, *J* = 9.9, 4.5 Hz, 1H), 2.67 – 2.62 (m, 1H), 2.50 – 2.44 (m, 1H), 2.37 (td, *J* = 12.8, 6.0 Hz, 1H), 2.11-2.06 (m, 1H), 1.73 – 1.55 (m, 4H), 1.26 – 1.23 (m, 1H). HPLC analysis (chiralpak AD-H column, hexane:2-propanol = 90:10, flow rate = 1 mL/min, wavelength = 238 nm), t (major) = 11.48 min, t (minor) = 16.90 min.

(*R*)-2-((*S*)-1-(4-Bromophenyl)-2-nitroethyl)cyclohexan-1-one (3g)[2,4,5,7,8]

White solid, (0.060 g, 0.19 mmol, 93% yield), 92% *ee*, 87:13 *dr* (*syn: anti*). $[\alpha]_D^{20}$= +16.8 (c 0.16, $CHCl_3$). ^1H NMR (500 MHz, $CDCl_3$) δ 7.45 (d, *J* = 8.4 Hz, 2H), 7.06 (d, *J* = 8.4 Hz, 2H), 4.93 (dd, *J* = 12.6, 4.5 Hz, 1H), 4.60 (dd, *J* = 12.6, 10.0 Hz, 1H), 3.74 (td, *J* = 9.9, 4.5 Hz, 1H), 2.67 – 2.61 (m, 1H), 2.49 – 2.44 (m, 1H), 2.37 (td, *J* = 12.8, 6.0 Hz, 1H), 2.11 – 2.06 (m, 1H), 1.72 – 1.55 (m, 4H), 1.26 – 1.19 (m, 1H).HPLC analysis (chiralpak AD-H column, hexane:2-propanol = 90:10, flow rate = 1 mL/min, wavelength = 220 nm), t (major) = 12.42 min, t (minor) = 19.45 min.

(*R*)-2-((*S*)-1-(3-Methoxyphenyl)-2-nitroethyl)cyclohexan-1-one (3h)[6]

White solid, (0.051 g, 0.18 mmol, 92% yield), 94% *ee*, 92:8 *dr* (*syn*: *anti*). $[\alpha]_D^{20}$= +31.9 (c 0.15, $CHCl_3$). ^1H NMR (500 MHz, $CDCl_3$) δ 7.23 (t, *J* = 7.9 Hz, 1H), 6.81 – 6.78 (m, 1H), 6.77 – 6.73 (m, 1H), 6.70 (t, *J* = 2.1 Hz, 1H), 4.92 (dd, *J* = 12.5, 4.5 Hz, 1H), 4.61 (dd, *J* = 12.5, 10.0 Hz, 1H), 3.79 (s, 3H), 3.72 (td, *J* = 10.0, 4.5 Hz, 1H), 2.69 – 2.63 (m, 1H), 2.52 – 2.44 (m, 1H), 2.38 (tdd, *J* = 12.8, 5.9, 1.1 Hz, 1H), 2.14 – 2.04 (m, 1H), 1.69 – 1.59 (m, 4H), 1.26 – 122 (m, 1H). HPLC analysis (chiralpak AD-H column, hexane:2-propanol = 95:5, flow rate = 1 mL/min, wavelength = 254 nm), t (major) = 31.62 min, t (minor) = 34.11 min.

(*R*)-2-((*S*)-1-(3-Fluorophenyl)-2-nitroethyl)cyclohexan-1-one (3i)[8]

White solid, (0.049 g, 0.19 mmol, 93% yield), 95% *ee*, >99:1 *dr* (*syn*: *anti*). $[\alpha]_D^{20}$= +27.4 (c 0.54, $CHCl_3$). ^1H NMR (500 MHz, $CDCl_3$) δ 7.25 – 7.21 (m, 1H), 6.93 – 6.89 (m, 2H), 6.85 – 6.83 (m, 1H), 4.89 (dd, *J* = 12.7, 4.4 Hz, 1H), 4.55 (dd, *J* = 12.8, 10.0 Hz, 1H), 3.72 (td, *J* = 10.0, 4.5 Hz, 1H), 2.63 – 2.58 (m, 1H), 2.45 – 2.38 (m, 1H), 2.37 – 2.29 (m, 1H), 2.07 – 2.00 (m, 1H), 1.71 – 1.48 (m, 4H), 1.23 – 1.17 (m, 1H). HPLC analysis (chiralpak AD-H column, hexane:2-propanol = 95:5, flow rate = 1 mL/min, wavelength = 254 nm), t (major) = 14.59 min, t (minor) = 16.68 min.

(*R*)-2-((*S*)-1-(3-Bromophenyl)-2-nitroethyl)cyclohexan-1-one (3j)[6]

White solid, (0.062 g, 0.19 mmol, 95% yield), 94% *ee*, >99:1 *dr* (*syn: anti*). $[\alpha]_D^{20}$= +48.4 (c 0.43, $CHCl_3$).^1H NMR (500 MHz, $CDCl_3$) δ 7.41 (dd, *J* = 7.9, 1.8 Hz, 1H), 7.33 (d, *J* = 2.1 Hz, 1H), 7.20 (t, *J* = 7.8 Hz, 1H), 7.11 (d, *J* = 7.7 Hz, 1H), 4.93 (dd, *J* = 12.8, 4.4 Hz, 1H), 4.61 (dd, *J* = 12.8, 10.0 Hz, 1H), 3.74 (td, *J* = 9.9, 4.4 Hz, 1H), 2.68 – 2.62 (m, 1H), 2.48 (dt, *J* = 13.2, 3.9 Hz, 1H), 2.38 (td, *J* = 12.8, 5.9 Hz, 1H), 2.12 – 2.07 (m, 1H), 1.75 – 1.59 (m, 4H), 1.28 – 1.23 (m, 1H). HPLC analysis (chiralpak AD-H column, hexane:2-propanol = 95:5, flow rate = 1 mL/min, wavelength = 254 nm), t (major) = 15.79 min, t (minor) = 17.53 min.

(*R*)-2-((*S*)-1-(2-Methoxyphenyl)-2-nitroethyl)cyclohexan-1-one (3k)[3,6,7]

White solid, (0.052 g, 0.19 mmol, 93% yield), >99% *ee*, >99:1 *dr* (*syn: anti*). $[\alpha]_D^{20}$= +41.9 (c 0.21, $CHCl_3$). ^1H NMR (500 MHz, $CDCl_3$) δ 7.23 (dd, *J* = 7.8, 1.8 Hz, 1H), 7.08 (dd, *J* = 7.4, 1.7 Hz, 1H), 6.91 – 6.85 (m, 2H), 4.88 – 4.77 (m, 2H), 3.95 (td, *J* = 9.9, 4.9 Hz, 1H), 3.84 (s, 3H), 3.00 – 2.95 (m, 1H), 2.52 – 2.44 (m, 1H), 2.39 (tdd, *J* = 12.8, 5.9, 1.2 Hz, 1H), 2.09 – 2.04 (m, 1H), 1.69 – 1.56 (m, 4H), 1.25 – 1.18 (m, 1H). HPLC analysis (chiralpak IA-H column, hexane:2-propanol = 99:1, flow rate = 1 mL/min, wavelength = 220 nm), t (major) = 29.53 min.

(*R*)-2-((*S*)-1-(2-Fluorophenyl)-2-nitroethyl)cyclohexan-1-one (3l)[6]

White solid, (0.048 g, 0.18 mmol, 90% yield), 93% *ee*, 87:13 *dr* (*syn: anti)*. $[\alpha]_D^{20}$= +36.3 (c 0.13, $CHCl_3$). ^1H NMR (500 MHz, $CDCl_3$) δ 7.30 – 7.24 (m, 1H), 7.18 (td, *J* = 7.5, 1.8 Hz, 1H), 7.10 (td, *J* = 7.5, 1.2 Hz, 1H), 7.07 – 7.03 (m, 1H), 4.93 (dd, *J* = 12.7, 4.3 Hz, 1H), 4.71 (dd, *J* = 12.7, 10.2 Hz, 1H), 3.99 (td, *J* = 10.3, 4.3 Hz, 1H), 2.88 – 2.82 (m, 1H), 2.55 – 2.46 (m, 1H), 2.45 – 2.36 (m, 1H), 2.12 – 2.09 (m, 1H), 1.74 – 1.59 (m, 4H), 1.29 – 1.23 (m, 1H). HPLC analysis (chiralpak AD-H column, hexane:2-propanol = 98:2, flow rate = 1 mL/min, wavelength = 220 nm), t (major) = 20.42 min, t (minor) = 24.79 min.

(*R*)-2-((*S*)-1-(2-Chlorophenyl)-2-nitroethyl)cyclohexan-1-one (3m)[4,5]

White solid, (0.053 g, 0.19 mmol, 95% yield), >99% *ee*, 80:20 *dr (syn*: *anti*). $[\alpha]_D^{20}$= +46.4 (c

0.56, $CHCl_3$).^1H NMR (500 MHz, $CDCl_3$) δ 7.29 – 7.24 (m, 1H), 7.18 (td, *J* = 7.5, 1.8 Hz, 1H), 7.10 (td, *J* = 7.5, 1.2 Hz, 1H), 7.07 – 7.03 (m, 1H), 4.93 (dd, *J* = 12.7, 4.3 Hz, 1H), 4.71 (dd, *J* = 12.7, 10.2 Hz, 1H), 3.99 (td, *J* = 10.3, 4.3 Hz, 1H), 2.90 – 2.81 (m, 1H), 2.53 – 2.46 (m, 1H), 2.43 – 2.36 (m, 1H), 2.13 – 2.09 (m, 1H), 1.74 – 1.60 (m, 4H), 1.31 – 1.20 (m, 1H). HPLC analysis (chiralpak IA-H column, hexane:2-propanol = 95:5, flow rate = 1 mL/min, wavelength = 220 nm), t (major) = 11.76 min.

(*R*)-2-((*S*)-1-(2-Bromophenyl)-2-nitroethyl)cyclohexan-1-one (3n)[4,5,8]

White solid, (0.061 g, 0.19 mmol, 94% yield), 97% *ee*, >99:1 *dr* (*syn*: *anti*). $[\alpha]_D^{20}$= +63.6 (c 1.2, $CHCl_3$). ^1H NMR (500 MHz, $CDCl_3$) δ 7.57 (dd, *J* = 8.1, 1.3 Hz, 1H), 7.29 (td, *J* = 7.6, 1.3 Hz, 1H), 7.21 (dd, *J* = 7.8, 1.7 Hz, 1H), 7.13 (td, *J* = 7.7, 1.7 Hz, 1H), 4.94 – 4.86 (m, 2H), 4.31 (s, 1H), 2.90 (s, 1H), 2.56 – 2.45 (m, 1H), 2.39 (td, *J* = 12.8, 6.0 Hz, 1H), 2.13 – 2.08 (m, 1H), 1.77 – 1.57 (m, 4H), 1.42 – 1.36 (m, 1H). HPLC analysis (chiralpak AD-H column, hexane:2-propanol = 95:5, flow rate = 1 mL/min, wavelength = 254 nm), t (major) = 13.72 min, t (minor) = 21.64 min.

(*R*)-2-((*S*)-1-(2,4-Dichlorophenyl)-2-nitroethyl)cyclohexan-1-one (3o)[2,3]

White solid, (0.060 g, 0.19 mmol, 95% yield), 98% *ee*, >99:1 *dr* (*syn*: *anti*). $[\alpha]_D^{20}$= +53.4 (c 0.36, $CHCl_3$). ^1H NMR (500 MHz, $CDCl_3$) δ 7.41 (d, *J* = 4.8 Hz, 1H), 7.30 – 7.21 (m, 1H), 7.18 (d, *J* = 8.4 Hz, 1H), 4.88 (d, *J* = 6.9 Hz, 2H), 4.25 (q, *J* = 7.0 Hz, 1H), 2.87 (s,1H), 2.55 – 2.33 (m, 2H), 2.19 – 2.04 (m, 1H), 1.78 – 1.54 (m, 4H), 1.39 – 1.26 (m, 1H). HPLC analysis (chiralpak AD-H column, hexane:2-propanol = 95:5, flow rate = 1 mL/min, wavelength = 220 nm), t (major) = 12.63 min, t (minor) = 16.07 min.

(*R*)-2-((*S*)-1-(Naphthalen-2-yl)-2-nitroethyl)cyclohexan-1-one (3p)[6-8]

White solid, (0.055 g, 0.18 mmol, 92% yield), 92% *ee*, >99:1 *dr* (*syn*: *anti*). $[\alpha]_D^{20}$= +36.7 (c 0.21, $CHCl_3$). ^1H NMR (500 MHz, $CDCl_3$) δ 7.81 (td, *J* = 9.6, 9.0, 6.1 Hz, 3H), 7.63 (d, *J* = 1.7 Hz, 1H), 7.51 – 7.45 (m, 2H), 7.29 (dd, *J* = 8.4, 1.9 Hz, 1H), 5.03 (dd, *J* = 12.5, 4.5 Hz, 1H), 4.73 (dd, *J* = 12.6, 10.0 Hz, 1H), 3.95 (td, *J* = 10.0, 4.5 Hz, 1H), 2.81 – 2.75 (m, 1H), 2.52 – 2.48 (m, 1H), 2.41 (tdd, *J* = 12.8, 5.9, 1.1 Hz, 1H), 2.10 – 2.05 (m, 1H), 1.74 – 1.56 (m, 4H), 1.31 – 1.25 (m, 1H). HPLC analysis (chiralpak AD-H column, hexane:2-propanol = 98:2, flow rate = 1 mL/min, wavelength = 238 nm), t (major) = 29.41 min, t (minor) = 36.21 min.

(*R*)-2-((*R*)-2-Nitro-1-(thiophen-2-yl)ethyl)cyclohexan-1-one (3q)[7]

White solid, (0.047 g, 0.19 mmol, 93% yield), 92% ee, >99:1 dr (*syn*: *anti*). $[\alpha]_D^{20}$= +23.4 (c 0.31, $CHCl_3$). ^1H NMR (500 MHz, $CDCl_3$) δ 7.22 (dd, *J* = 5.1, 1.2 Hz, 1H), 6.93 (dd, *J* = 5.2, 3.5 Hz, 1H), 6.87 (dd, *J* = 3.5, 1.2 Hz, 1H), 4.89 (dd, *J* = 12.6, 4.7 Hz, 1H), 4.65 (dd, *J* = 12.6, 9.4 Hz, 1H), 4.13 (td, *J* = 9.2, 4.7 Hz, 1H), 2.71 – 2.63 (m, 1H), 2.51 – 2.45 (m, 1H), 2.42 – 2.33 (m, 1H), 2.12 – 2.07 (m, 1H), 1.86 – 1.82 (m, 1H), 1.71 – 1.59 (m, 3H), 1.36 – 1.28 (m, 1H). HPLC analysis (chiralpak AD-H column, hexane:2-propanol = 98:2, flow rate = 1 mL/min, wavelength = 254 nm), t (major) = 44.15 min, t (minor) = 51.07 min.

(*R*)-2-((*R*)-1-(Furan-2-yl)-2-nitroethyl)cyclohexan-1-one (3r)[2-7]

White solid, (0.041 g, 0.17 mmol, 87% yield), 78% *ee*, 87:13 *dr* (*syn: anti*). $[\alpha]_D^{20}$= +8.4 (c 0.2, $CHCl_3$). ^1H NMR (500 MHz, $CDCl_3$) δ 7.34 (d, *J* = 1.9 Hz, 1H), 6.35 – 6.25 (m, 1H), 6.17 (d, *J* = 3.2 Hz, 1H), 4.79 (dd, *J* = 12.5, 4.7 Hz, 1H), 4.66 (dd, *J* = 12.5, 9.4 Hz, 1H), 3.96 (td, *J* = 9.2, 4.7 Hz, 1H), 2.77 – 2.72 (m, 1H), 2.49 – 2.41 (m, 1H), 2.41 – 2.32 (m, 1H), 2.12 – 2.08 (m, 1H), 1.77 – 1.72 (m, 1H), 1.67 – 1.61 (m, 3H), 1.35 – 1.23 (m, 1H). HPLC analysis (chiralpak IA-H column, hexane:2-propanol = 98:2, flow rate = 1 mL/min, wavelength = 220 nm), t (major) = 34.42 min, t (minor) = 39.67 min.

(*S*)-5-Nitro-4-phenylpentan-2-one (4)[1-4,6-8]

White solid, (0.038 g, 0.1 mmol, 98% yield), 27% *ee*. $[\alpha]_D^{20}$= +3.9 (c 0.51, $CHCl_3$).^1H NMR (500 MHz, $CDCl_3$) δ 7.35 (dd, *J* = 8.2, 6.7 Hz, 2H), 7.32 – 7.27 (m, 1H), 7.26 – 7.22 (m, 2H), 4.71 (dd, *J* = 12.3, 6.8 Hz, 1H), 4.62 (dd, *J* = 12.3, 7.8 Hz, 1H), 4.03 (p, *J* = 7.1 Hz, 1H), 2.94 (d, *J* = 7.1 Hz, 2H), 2.14 (s, 3H). HPLC analysis (chiralpak AD-H column, hexane:2-propanol =

90:10, flow rate = 1 mL/min, wavelength = 254 nm), t (major) = 11.10 min, t (minor) = 11.93 min.

References

[1] T. Mandal, and C. G. Zhao. *Angew. Chem.*, 2008, **120**, 7828–7831.

[2] C.-L. Cao, M.-C. Ye, X.-L. Sun, and Y. Tang. *Org. Lett.*, 2006, **8**, 2901-2904.

[3] A. Lu, P. Gao, Y. Wu, Y. Wang, Z. Zhou, and C. Tang. *Org. Biomol. Chem.*, 2009, **7**, 3141-3147.

[4] R. Rani, and R. K. Peddinti. *Tetrahedron: Asymmetry*, 2010, **21**, 2487–2492.

[5] J. Agarwal, and R. K. Peddinti. *Tetrahedron Lett.*, 2011, **52**, 117–121.

[6] A. Ruiz-Olalla, M. de Gracia Retamosa, and F. P. Cossío. *J. Org. Chem.*, 2015, **80**, 5588−5599.

[7] J. H. Shim, B. K. Ahn, J. Y. Lee, H. S. Kim, and D.-C. Ha. *Catalysts*, 2021, **11**, 1004.

[8] D. Rani, M. Khera, N. Goel, and J. Agarwal. *Mol. Catal*., 2023, **545**, 113185.

3. NMR spectra of products **3a–3r**

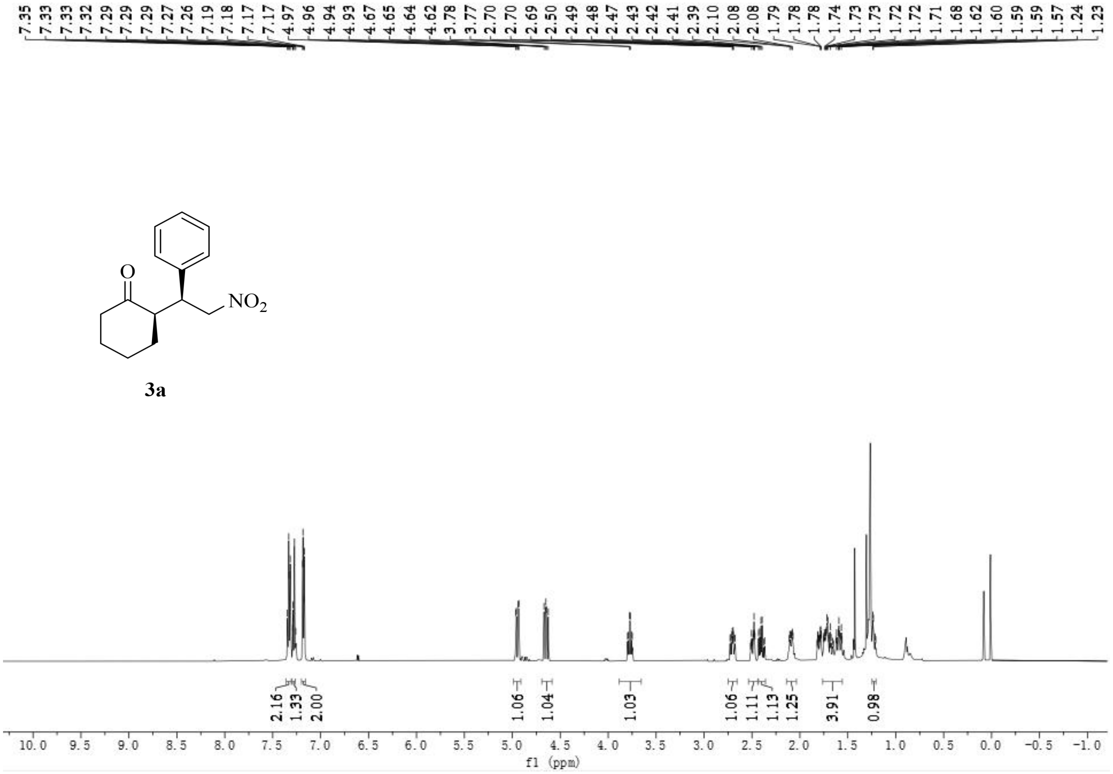

^{1}H NMR (500 MHz, $CDCl_3$) spectrum of compound **3a**

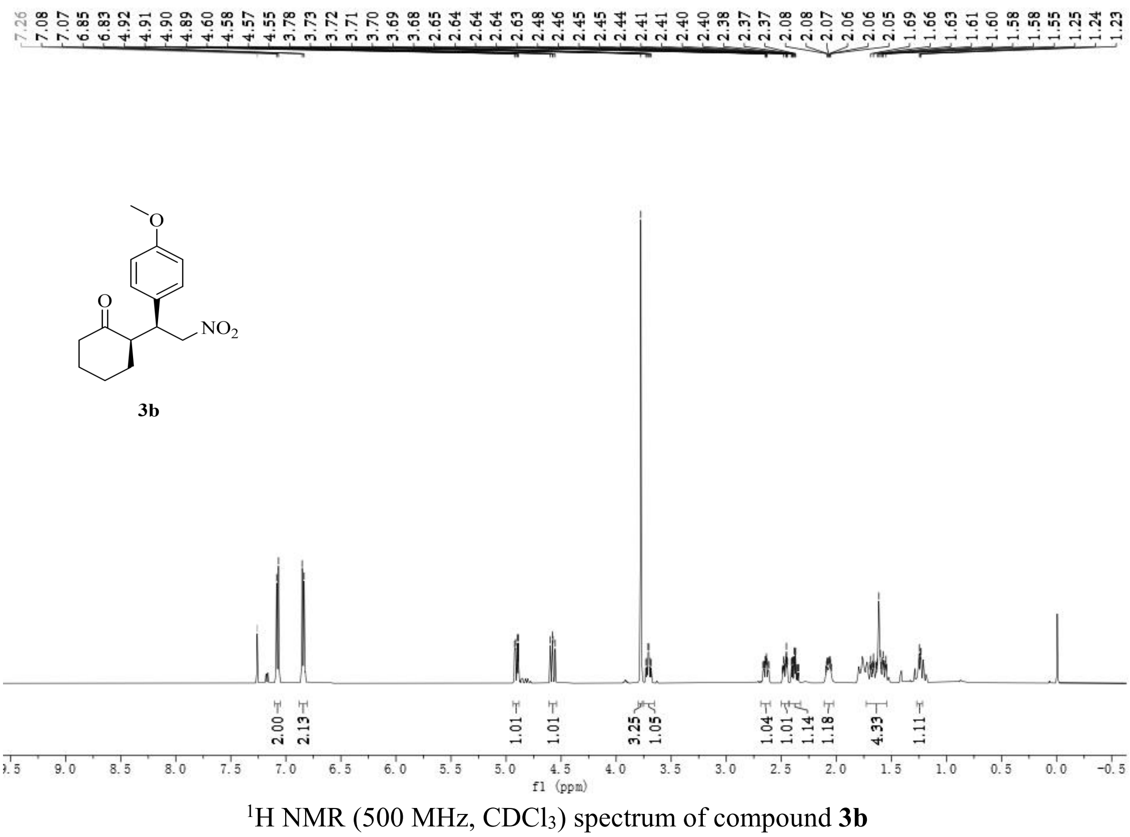

^{1}H NMR (500 MHz, $CDCl_3$) spectrum of compound **3b**

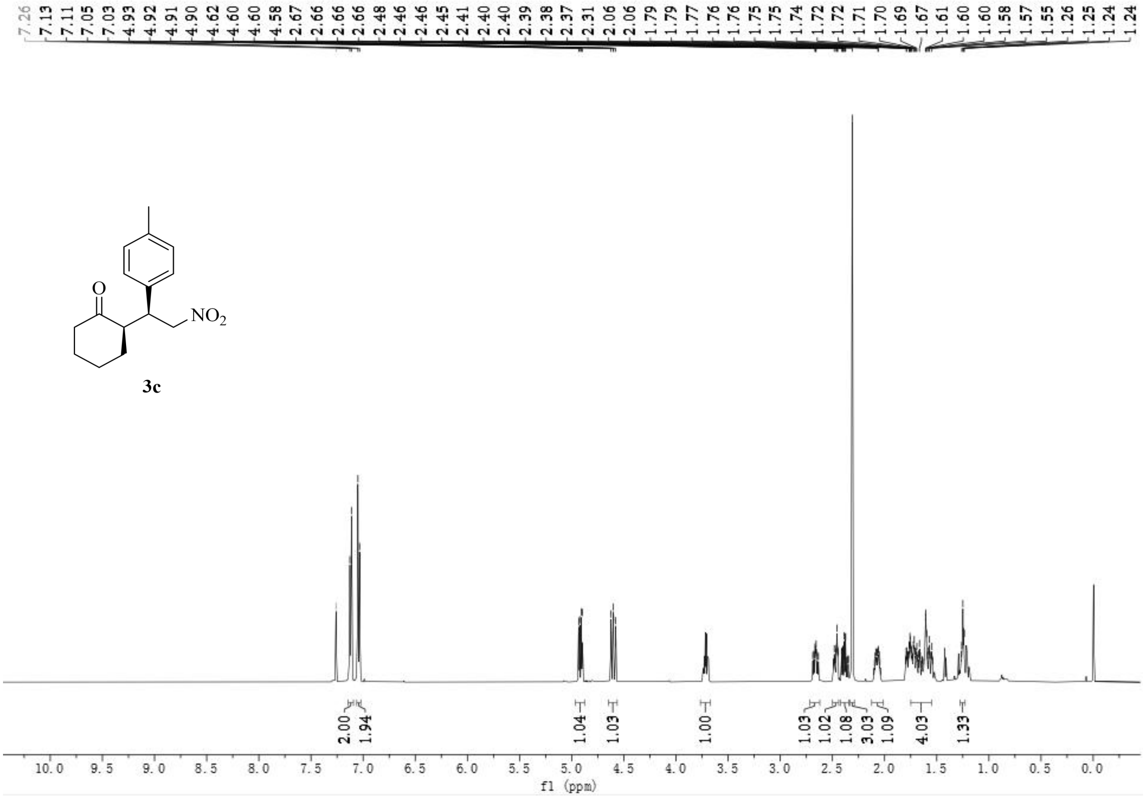

^{1}H NMR (500 MHz, $CDCl_3$) spectrum of compound **3c**

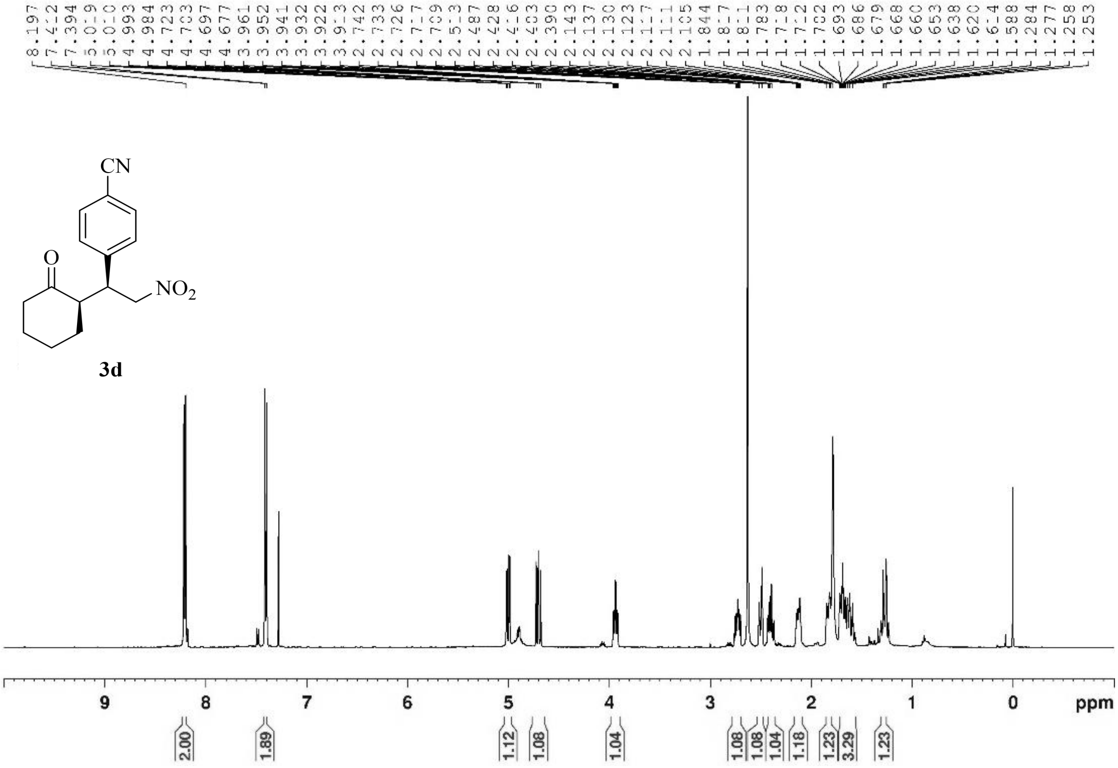

^{1}H NMR (500 MHz, $CDCl_3$) spectrum of compound **3d**

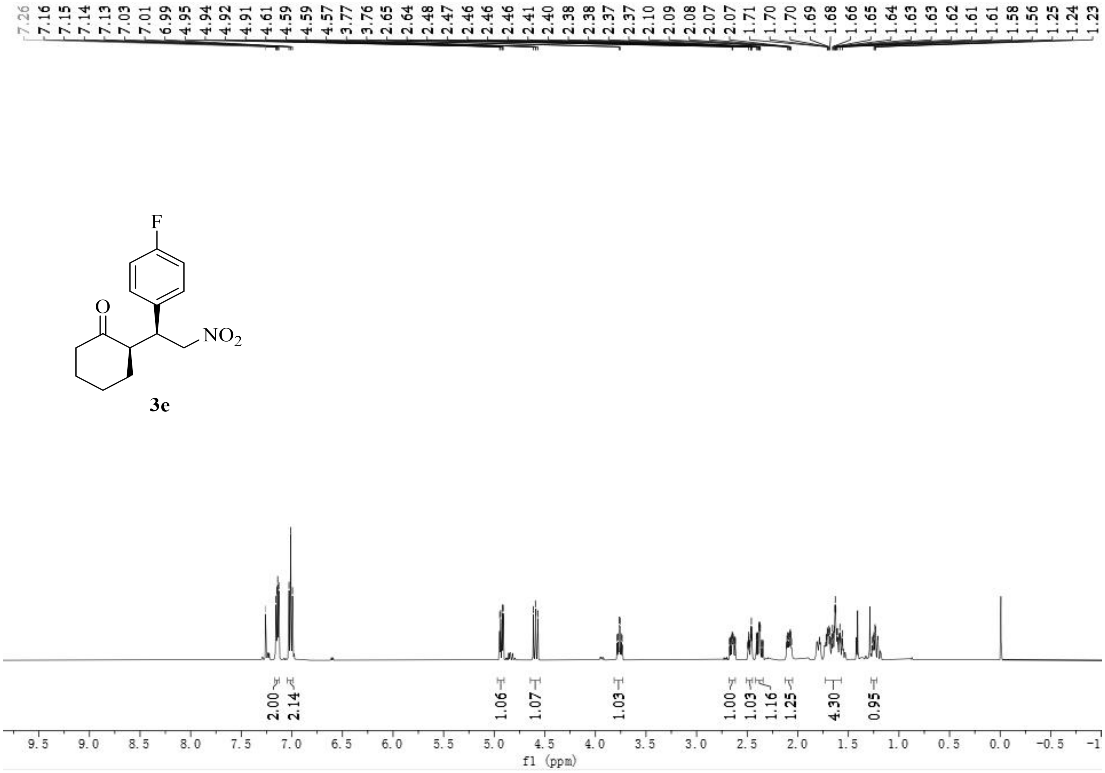

^{1}H NMR (500 MHz, $CDCl_3$) spectrum of compound **3e**

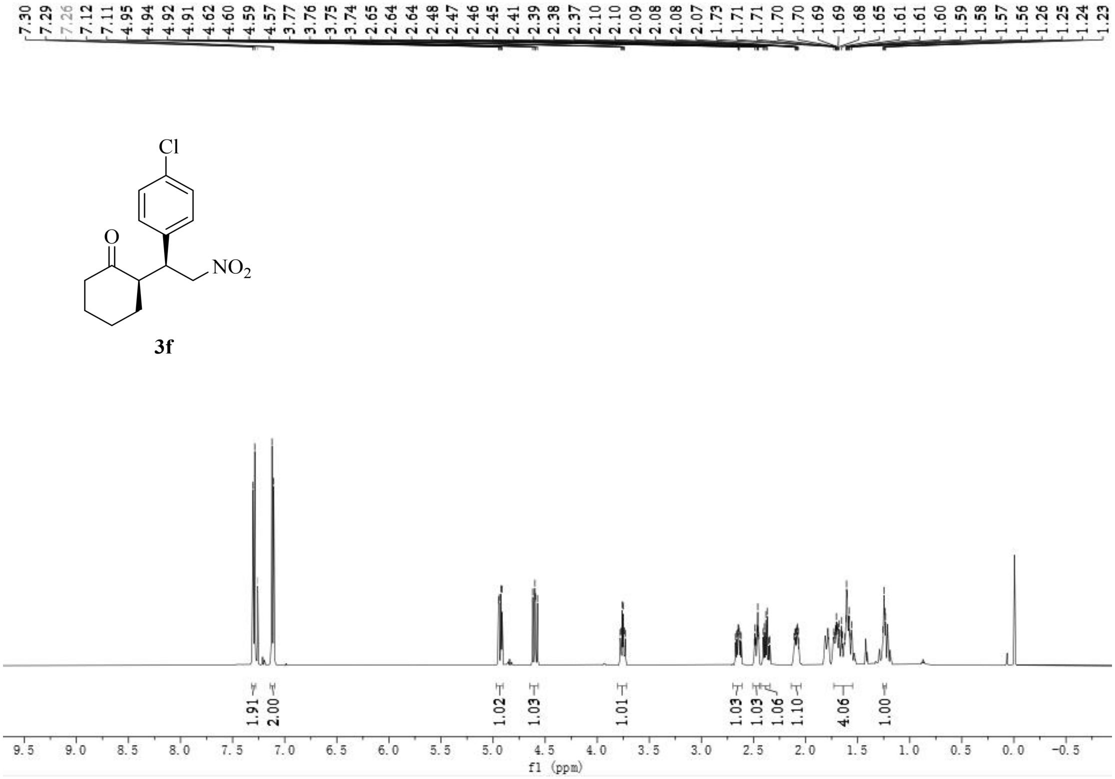

^{1}H NMR (500 MHz, $CDCl_3$) spectrum of compound **3f**

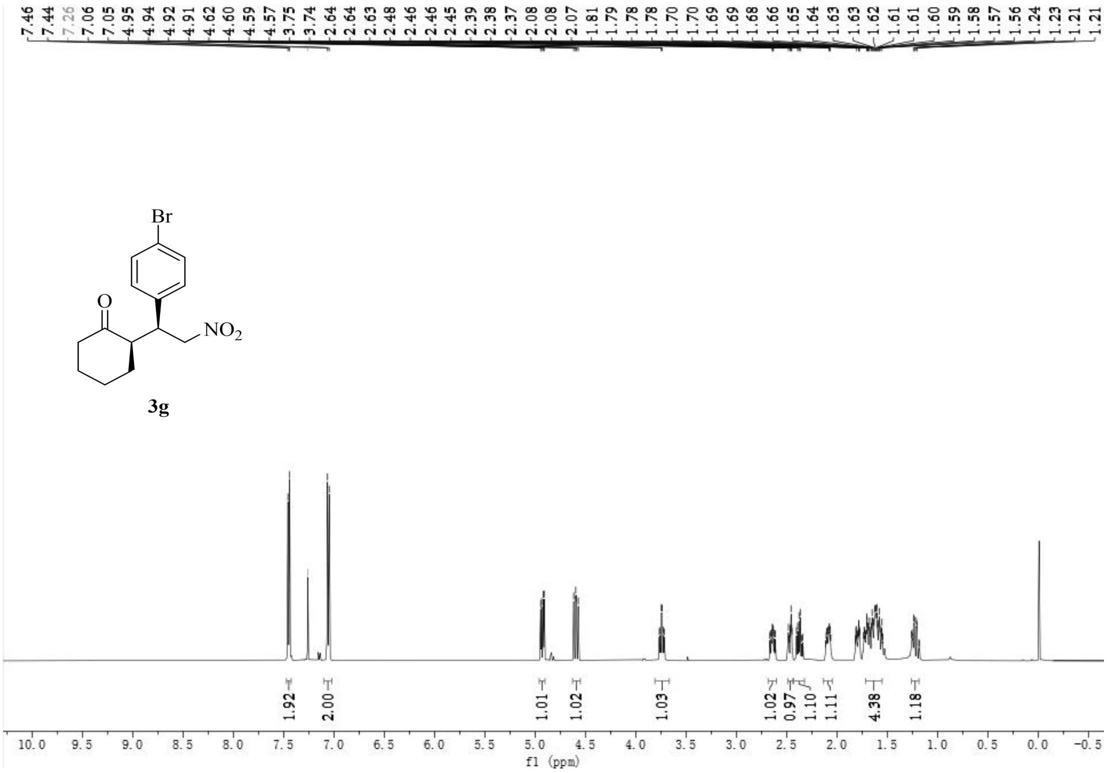

^{1}H NMR (500 MHz, CDCl3) spectrum of compound **3g**

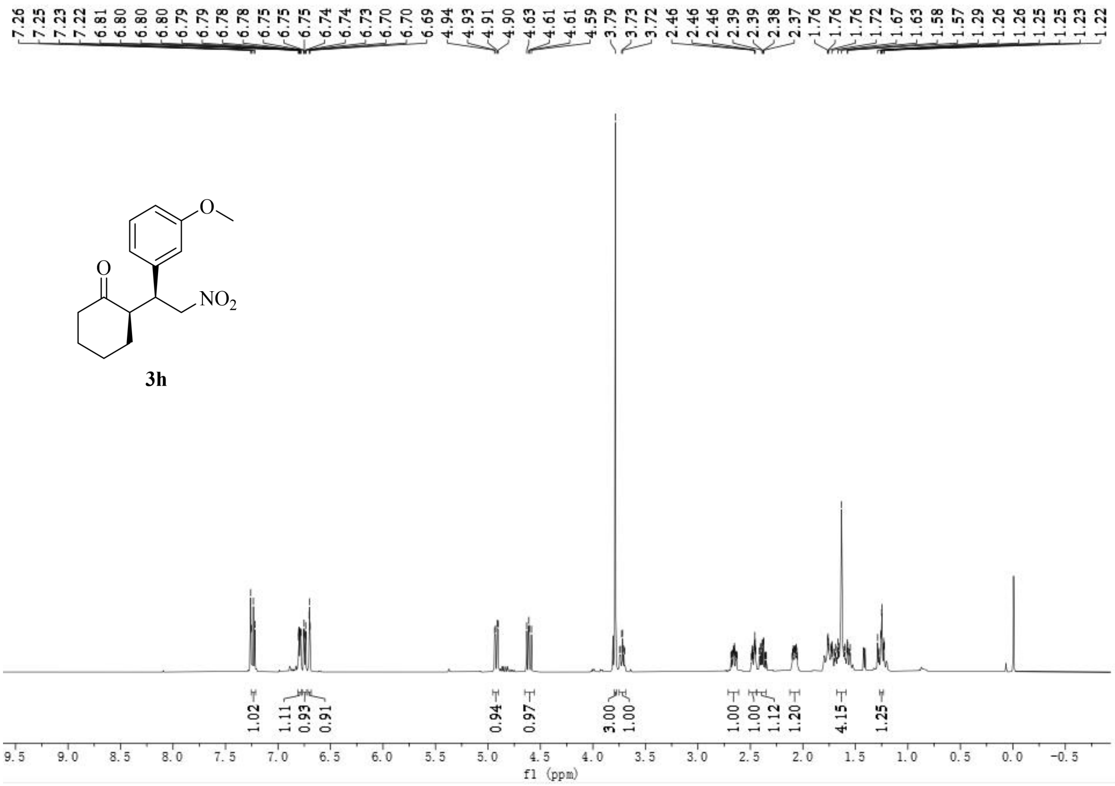

^{1}H NMR (500 MHz, CDCl3) spectrum of compound **3h**

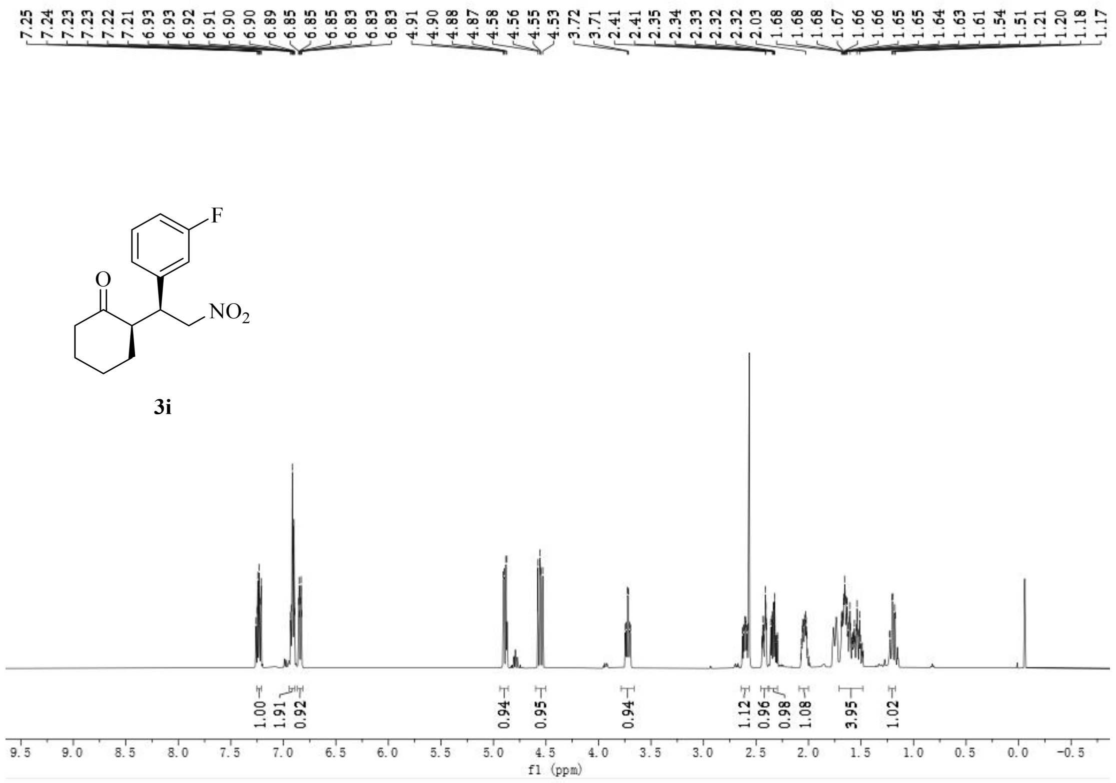

^{1}H NMR (500 MHz, $CDCl_3$) spectrum of compound **3i**

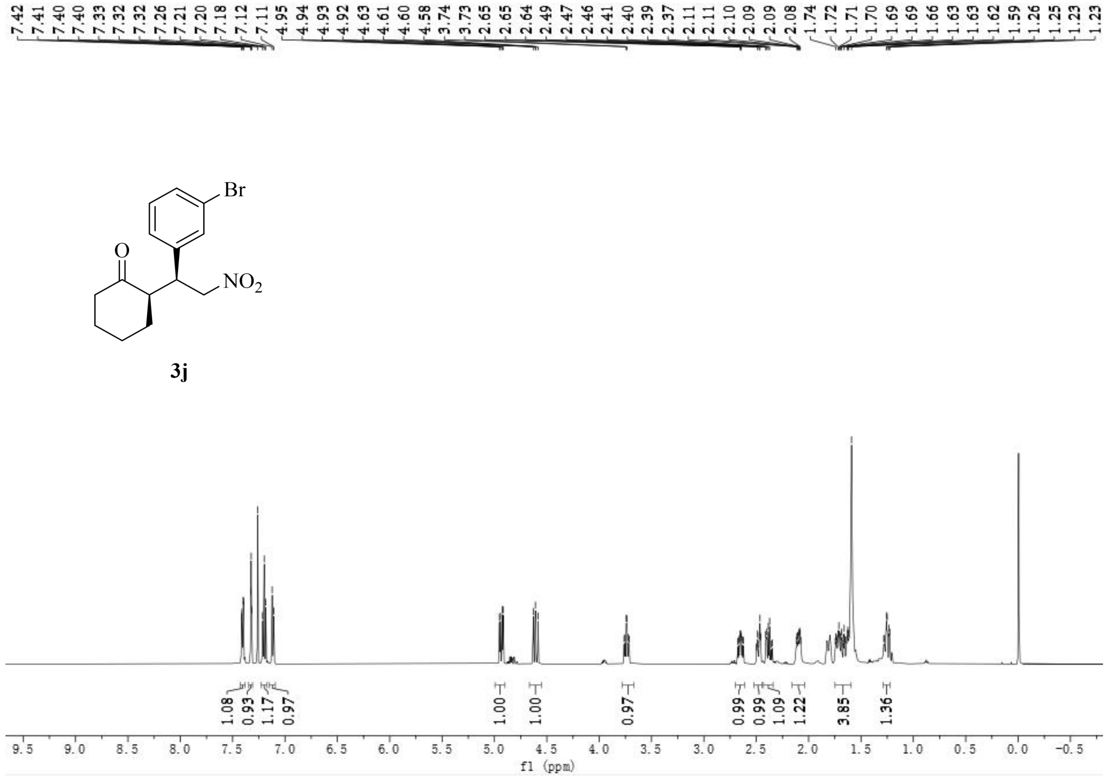

^{1}H NMR (500 MHz, $CDCl_3$) spectrum of compound **3j**

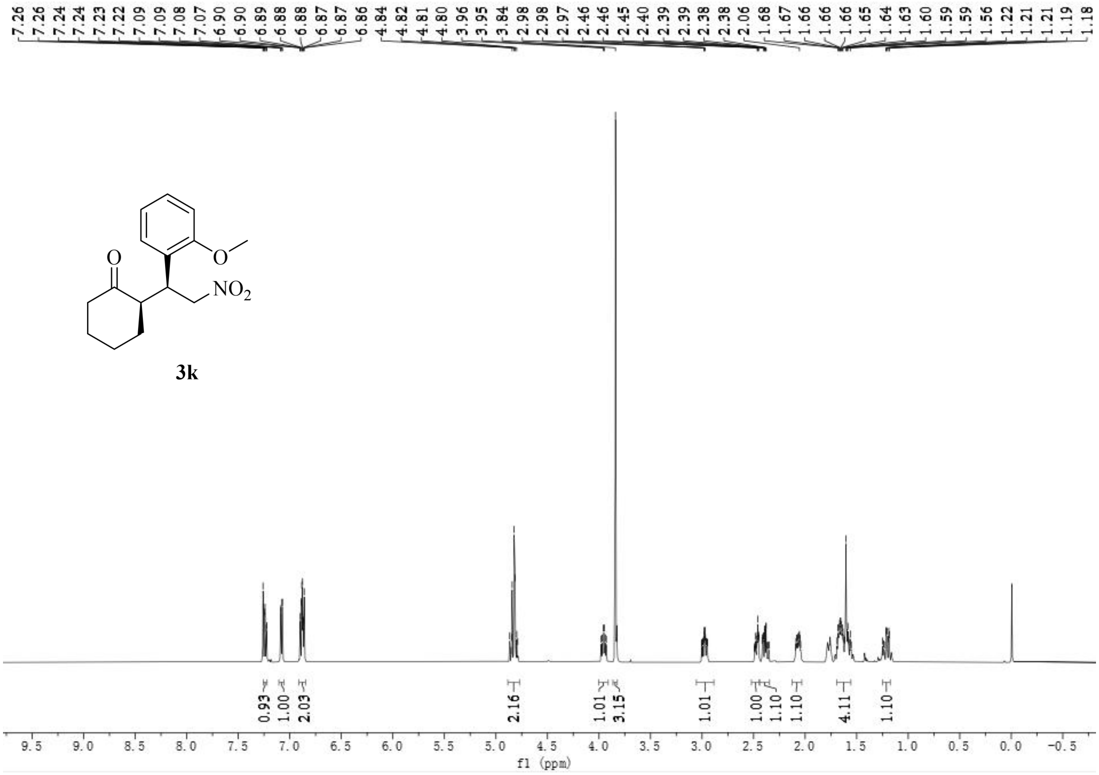

^{1}H NMR (500 MHz, $CDCl_3$) spectrum of compound **3k**

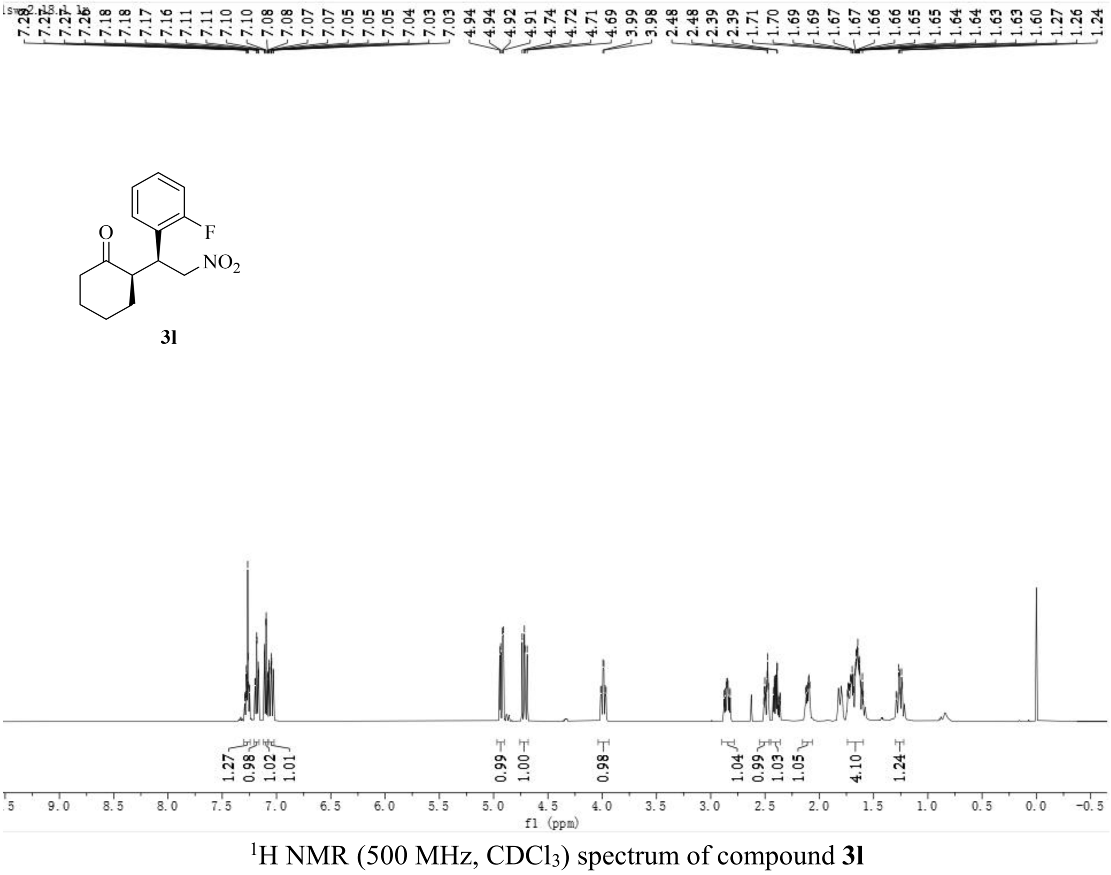

^{1}H NMR (500 MHz, $CDCl_3$) spectrum of compound **3l**

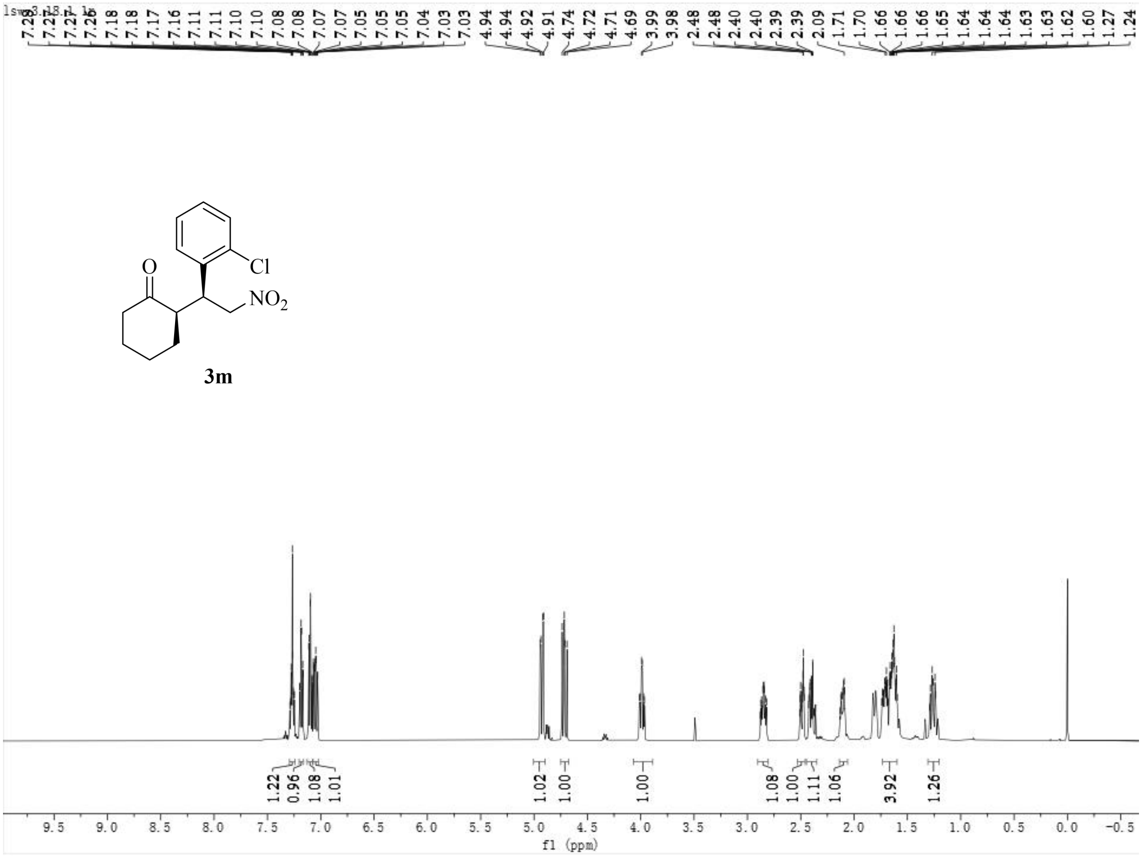

^1H NMR (500 MHz, $CDCl_3$) spectrum of compound **3m**

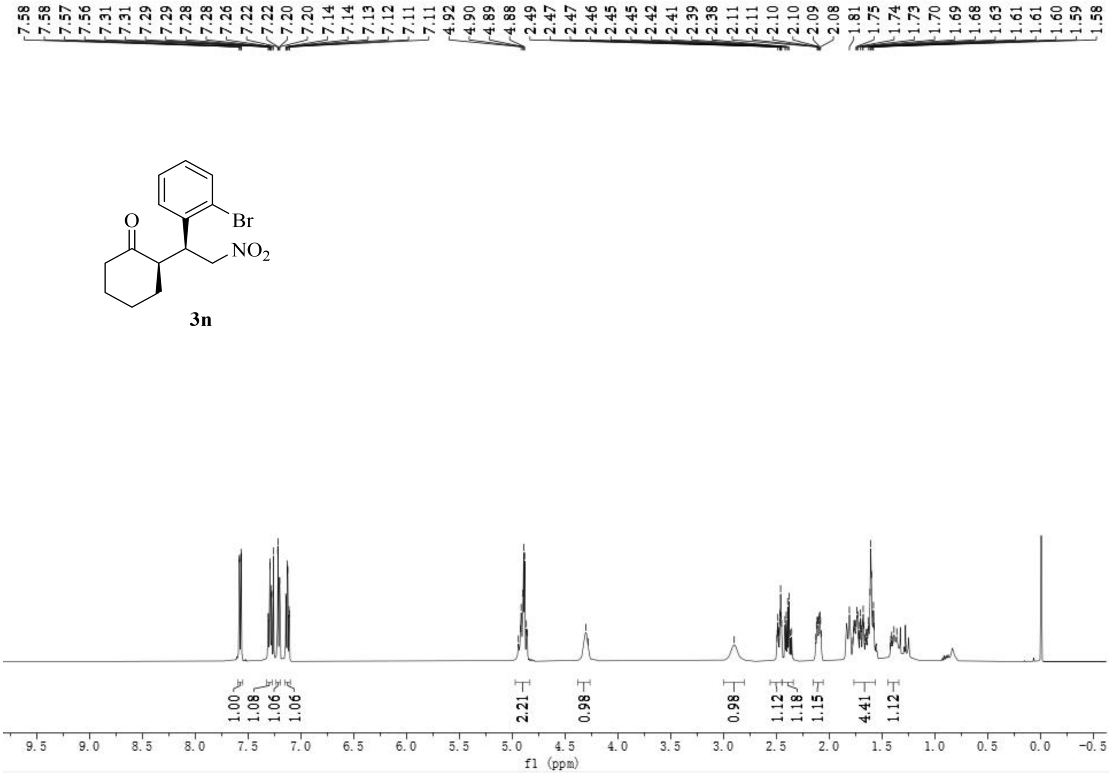

^1H NMR (500 MHz, $CDCl_3$) spectrum of compound **3n**

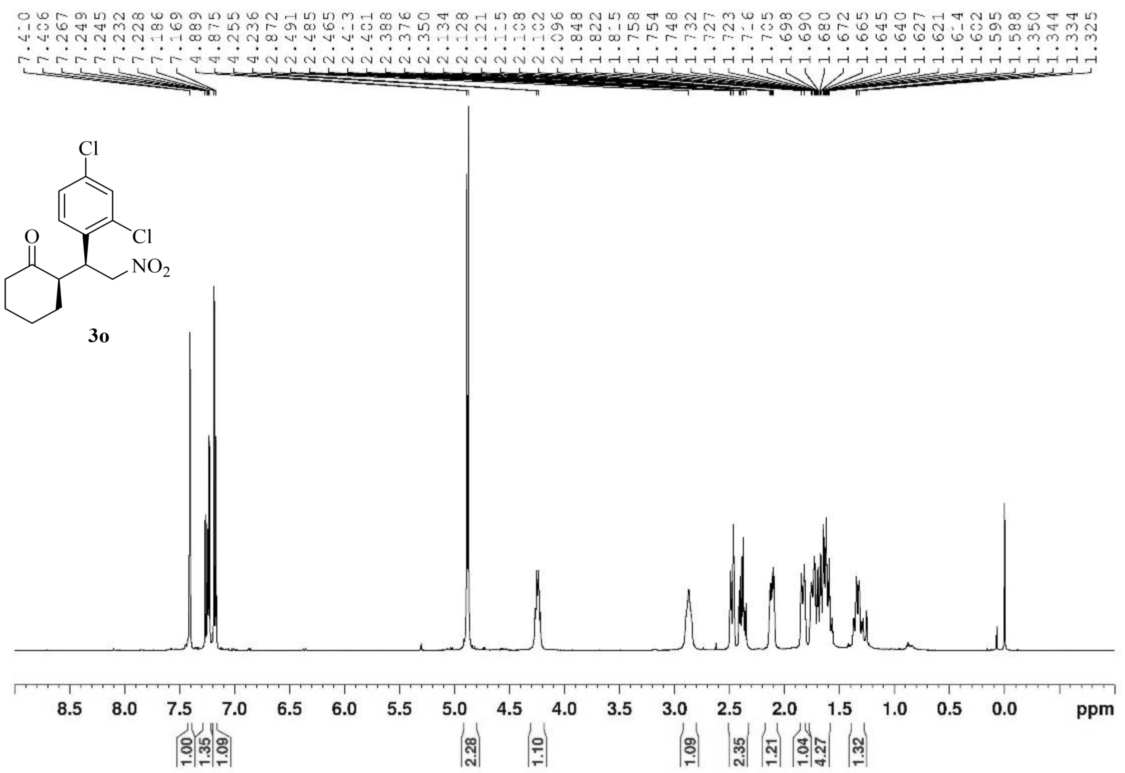

^{1}H NMR (500 MHz, $CDCl_3$) spectrum of compound **3o**

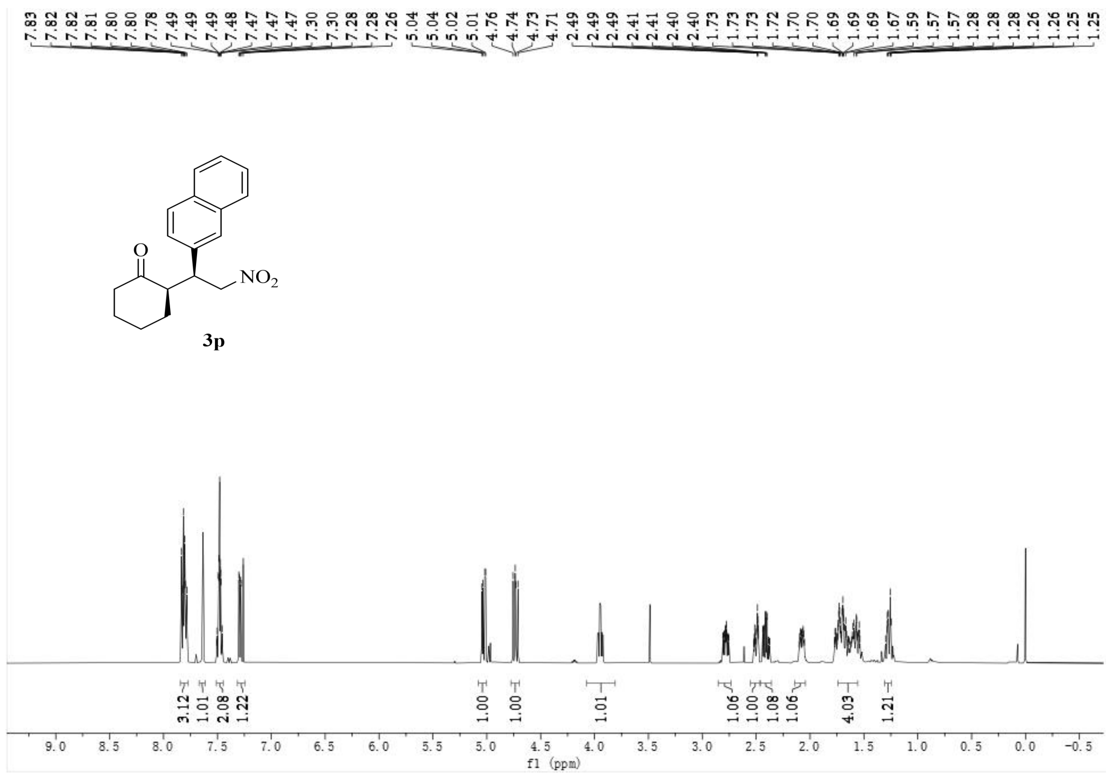

^{1}H NMR (500 MHz, $CDCl_3$) spectrum of compound **3p**

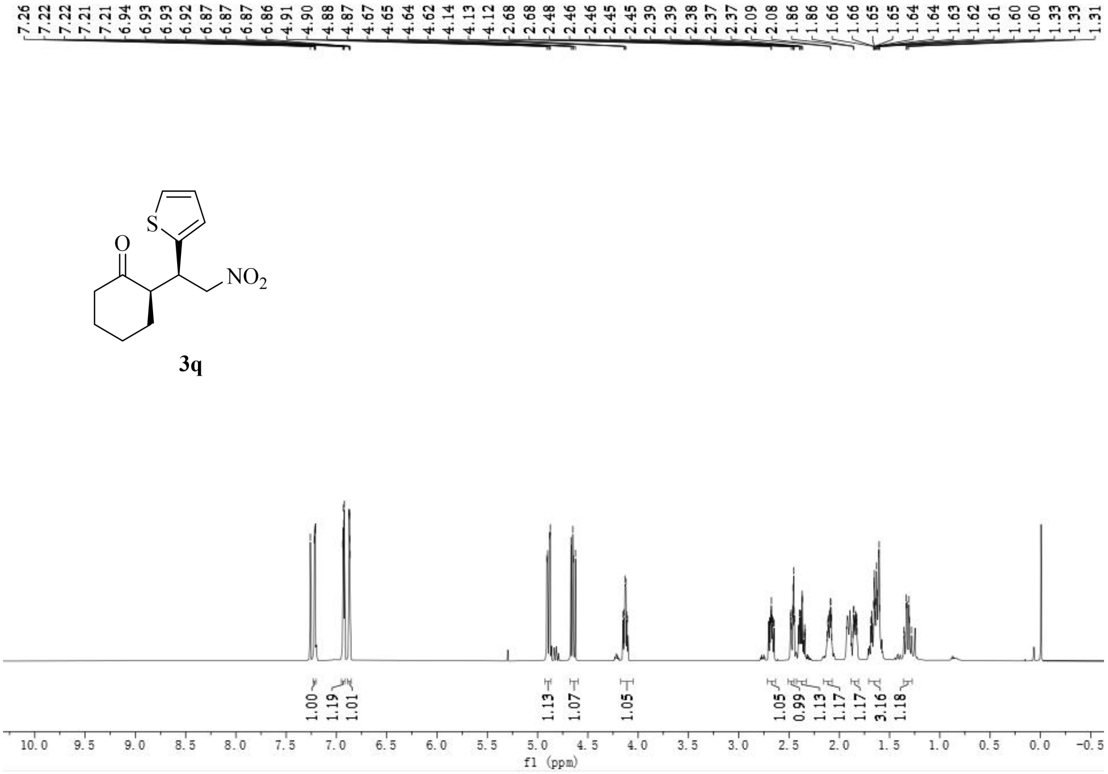

3q

^{1}H NMR (500 MHz, $CDCl_3$) spectrum of compound **3q**

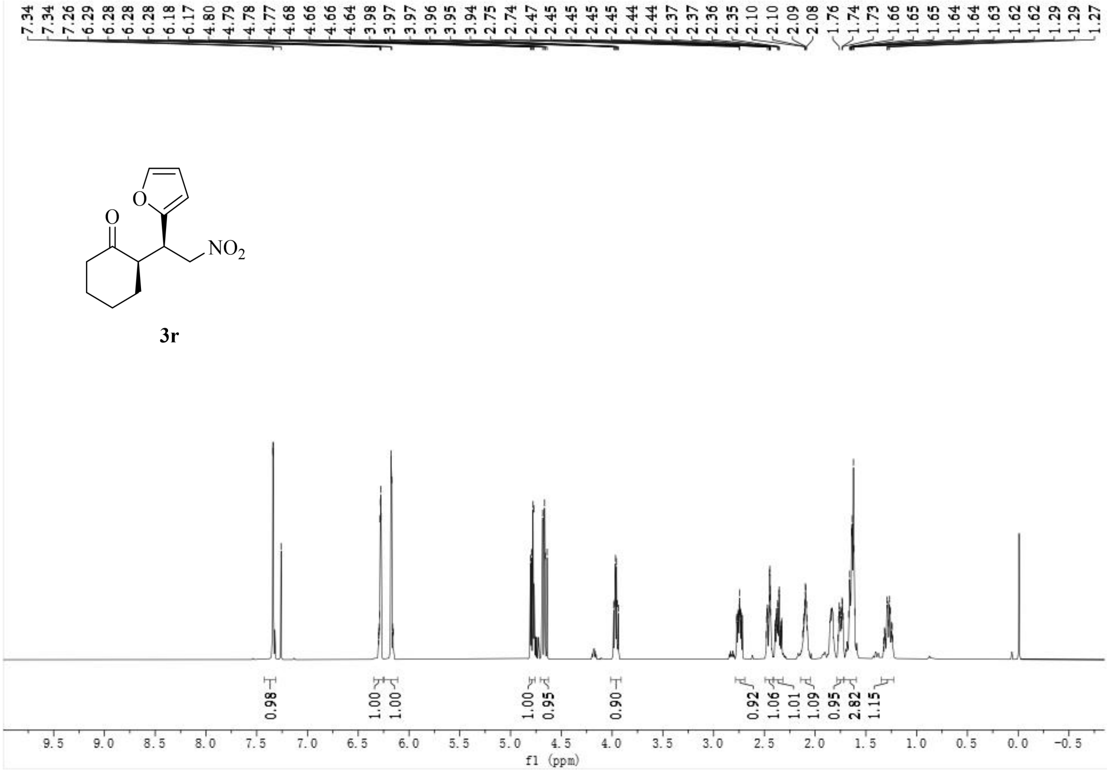

3r

^{1}H NMR (500 MHz, $CDCl_3$) spectrum of compound **3r**

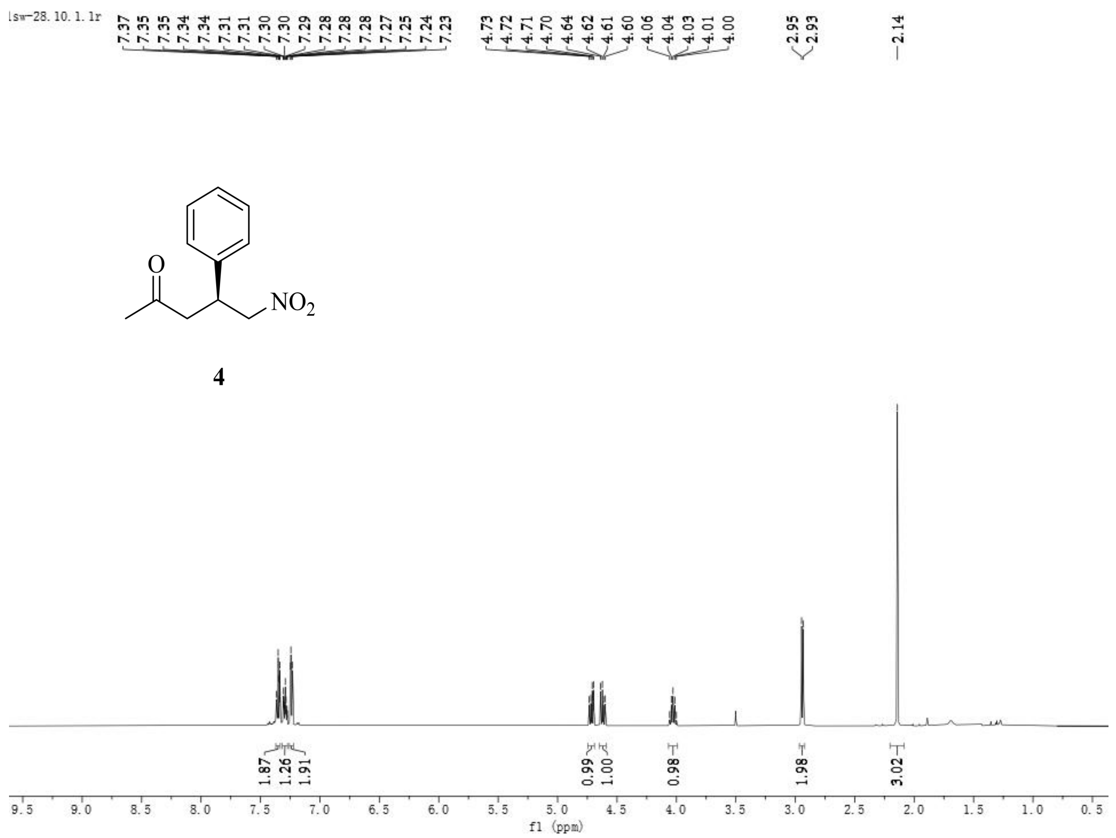

^{1}H NMR (500 MHz, $CDCl_3$) spectrum of compound **4**

4. HPLC traces of racemic and chiral products **3a–3r** and **4**

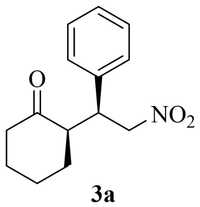

3a

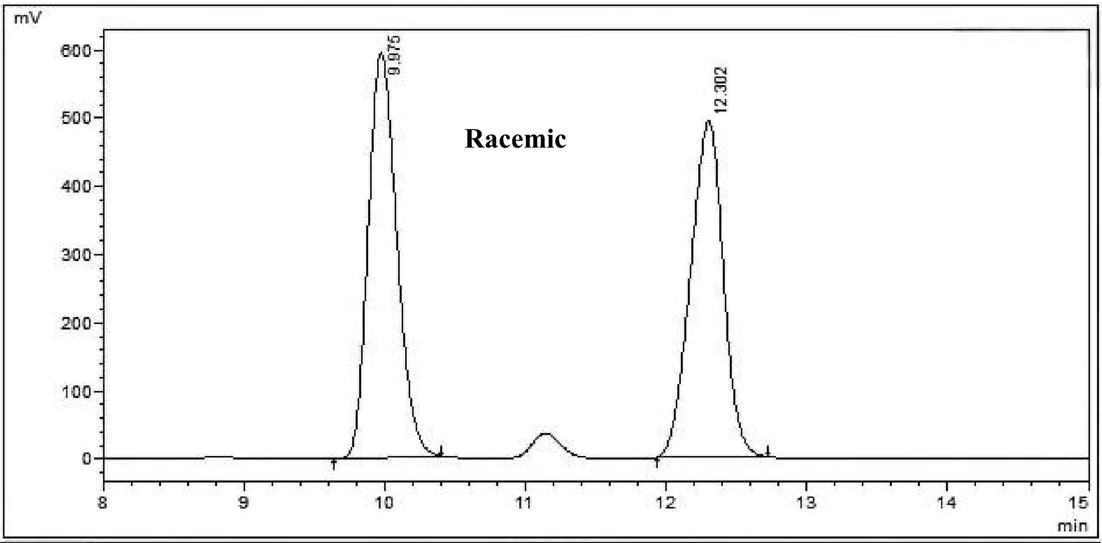

Ch1 220nm

	Ret. time (min)	Height (mv)	Width (min)	Area (mv*min)	Area %
1	9.975	592798	0.378	8361366	50.958
2	12.302	493199	0.441	8047130	49.042

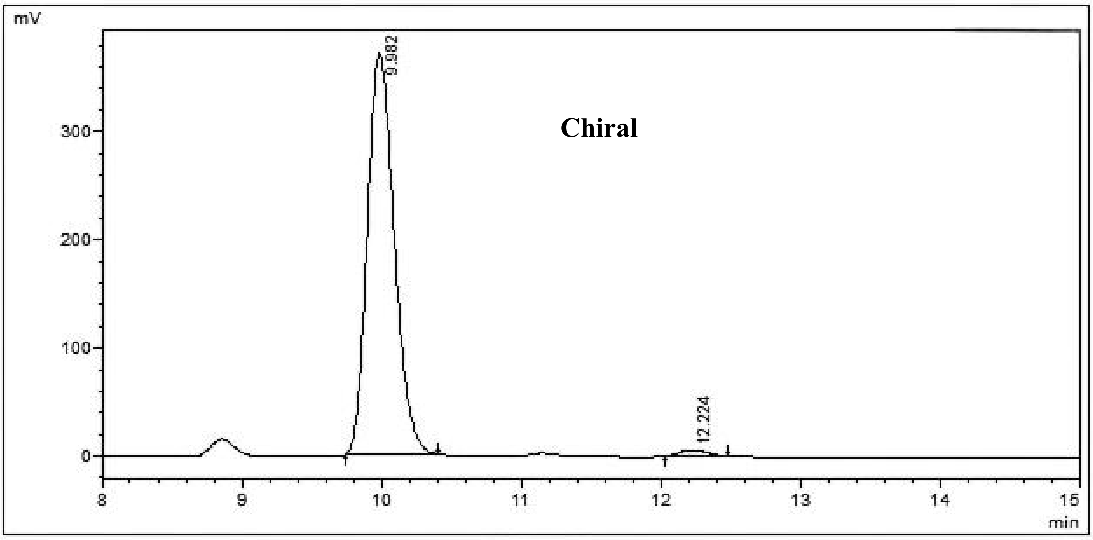

Ch1 220nm

	Ret. time (min)	Height (mv)	Width (min)	Area (mv*min)	Area %
1	9.982	371060	0.359	4968204	98.418
2	12.224	5834	0.380	79838	1.582

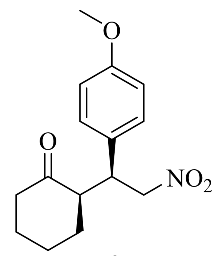

3b

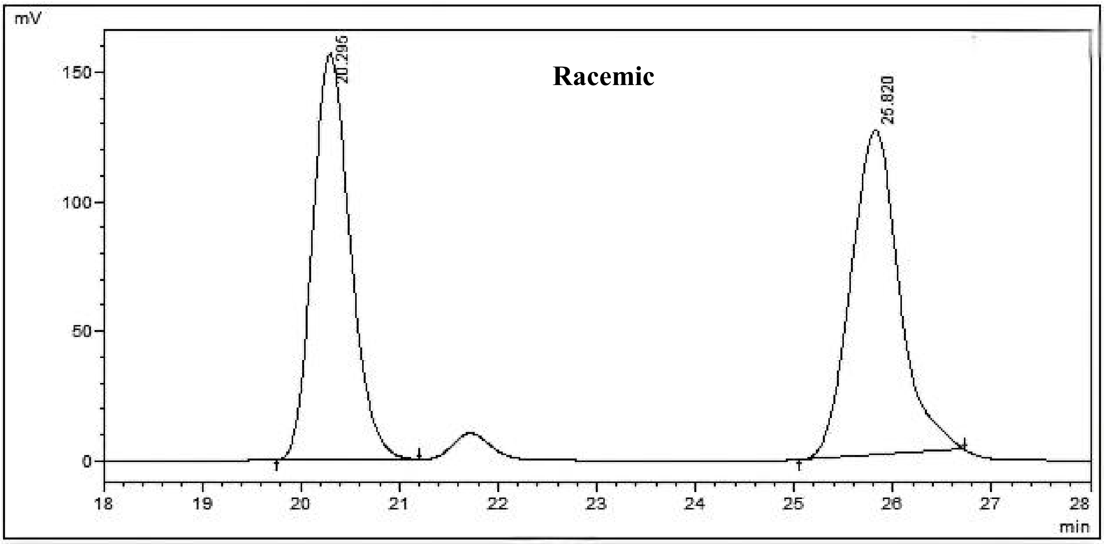

Ch1 238nm

	Ret. time (min)	Height (mv)	Width (min)	Area (mv*min)	Area %
1	20.295	156907	0.700	4170882	49.367
2	25.820	125556	0.877	4277803	50.633

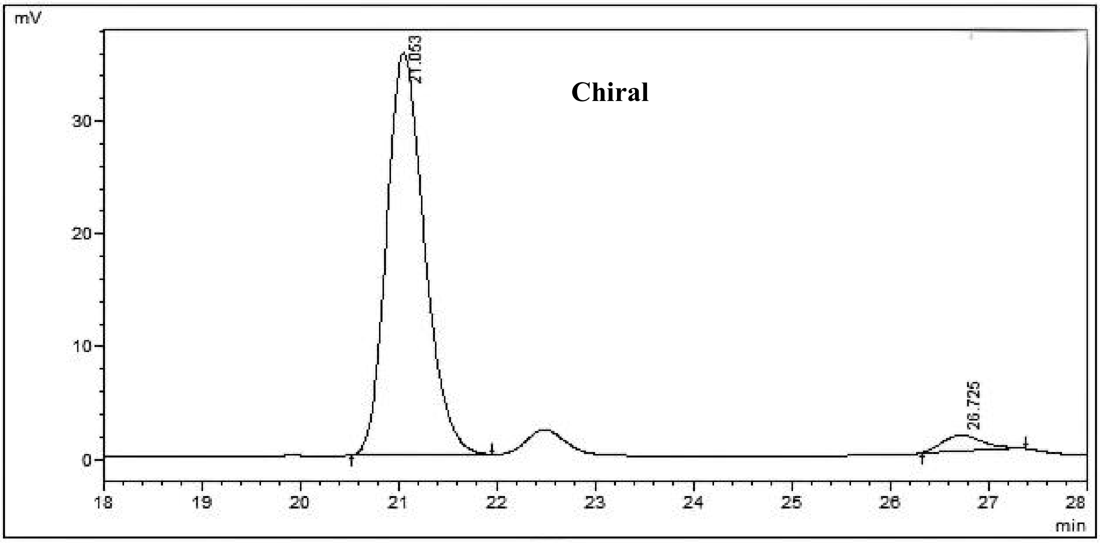

Ch1 238nm

	Rrt. time	Height (mv)	Width (min)	Area (mv*min)	Area %
1	21.053	35623	0.706	955646	95.896
2	26.725	1396	0.762	40902	4.104

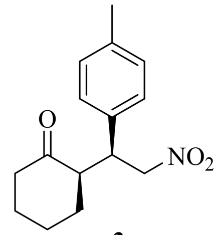

3c

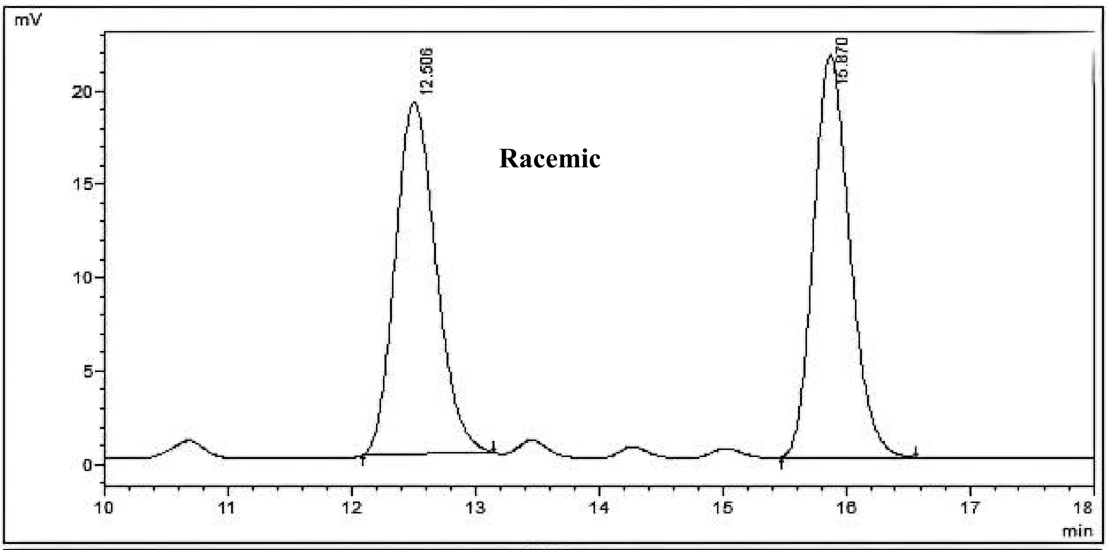

Ch1 254nm

	Ret. time (min)	Height (mv)	Width (min)	Area (mv*min)	Area%
1	12.506	18831	0.610	433064	49.689
2	15.870	21531	0.539	438488	50.311

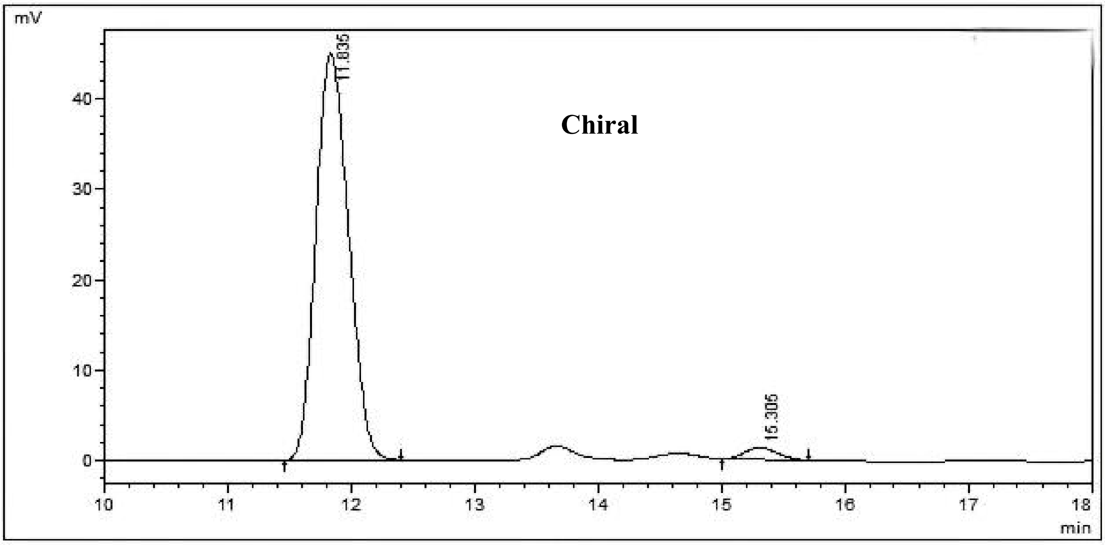

Ch1 254nm

	Ret. time (min)	Height (mv)	Width (min)	Area (mv*min)	Area %
1	11.835	44961	0.502	826763	97.133
2	15.305	1302	0.513	24404	2.867

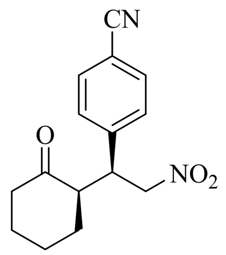

3d

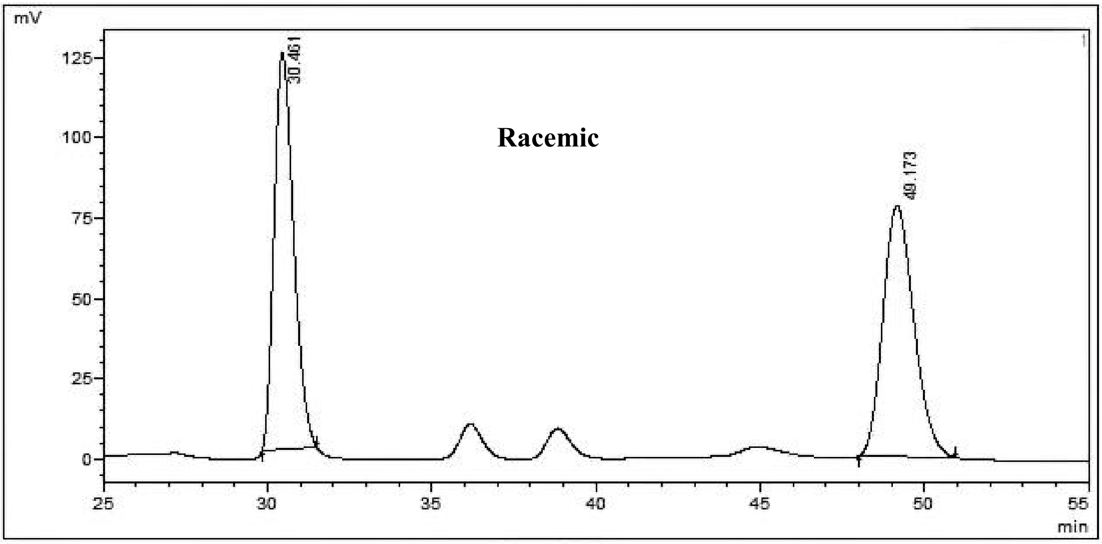

Ch1 220nm

	Ret. time (min)	Height (mv)	Width (min)	Area (mv*min)	Area %
1	30.461	123254	1.103	5095665	49.970
2	49.173	78208	1.724	5101777	50.030

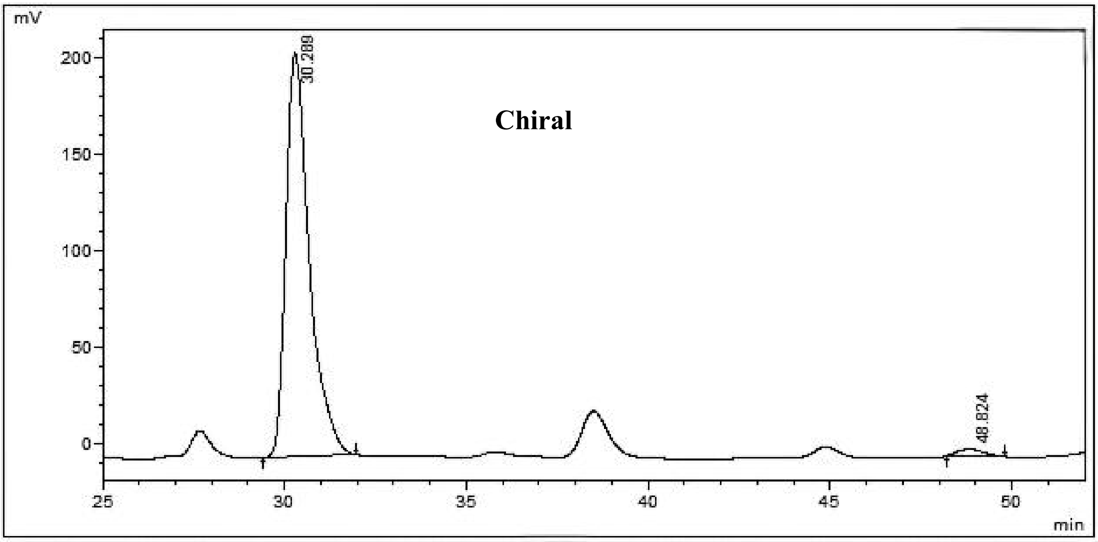

Ch1 220nm

	Ret. time (min)	Height (mv)	Width (min)	Area (mv*min)	Area %
1	30.289	209133	1.120	9412429	98.225
2	48.824	3471	1.342	170129	1.775

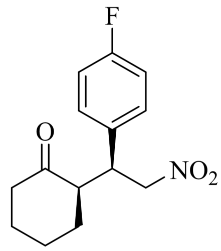

3e

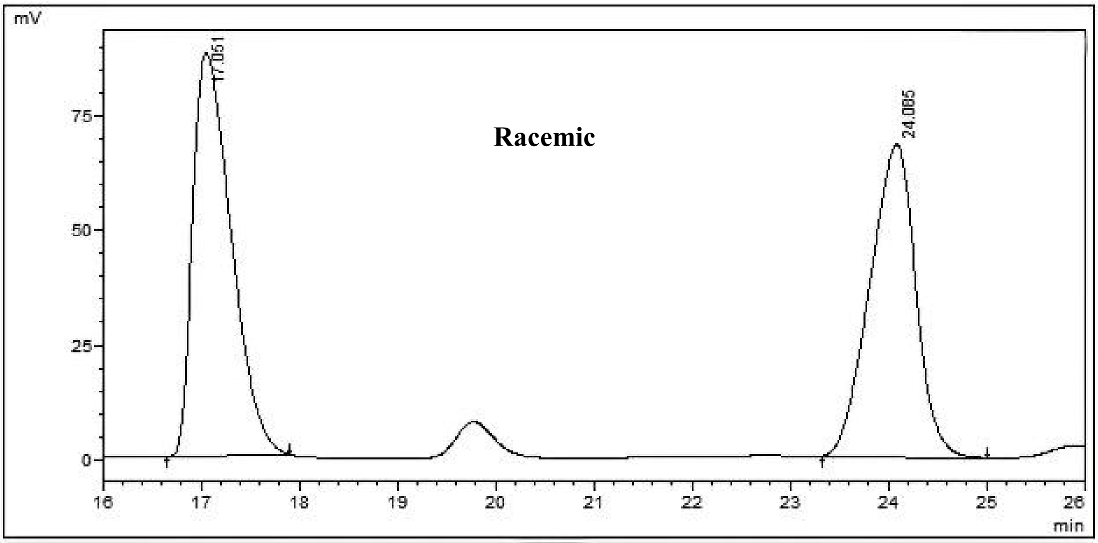

Ch1 254nm

	Ret. time (min)	Height (mv)	Width (min)	Area (mv*min)	Area %
1	17.051	56.309	0.735	2410630	51.942
2	24.085	43.691	0.884	2230391	48.058

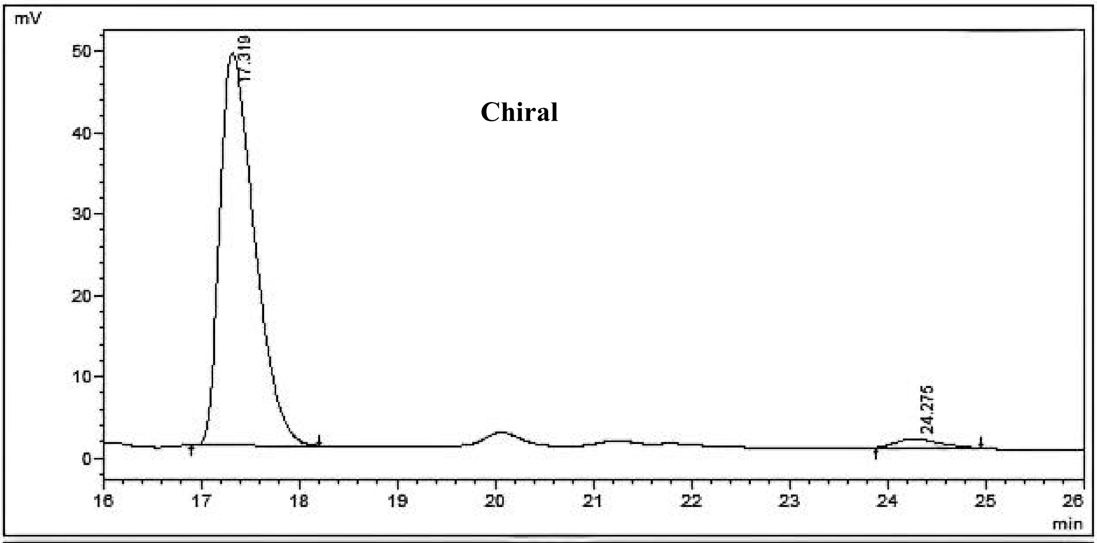

Ch1 254nm

	Ret. time (min)	Height (mv)	Width (min)	Area (mv*min)	Area %
1	17.319	48227	0.659	1190533	97.175
2	24.275	1113	0.830	34609	2.825

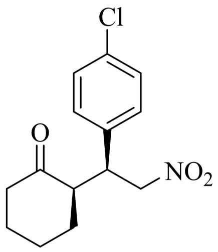

3f

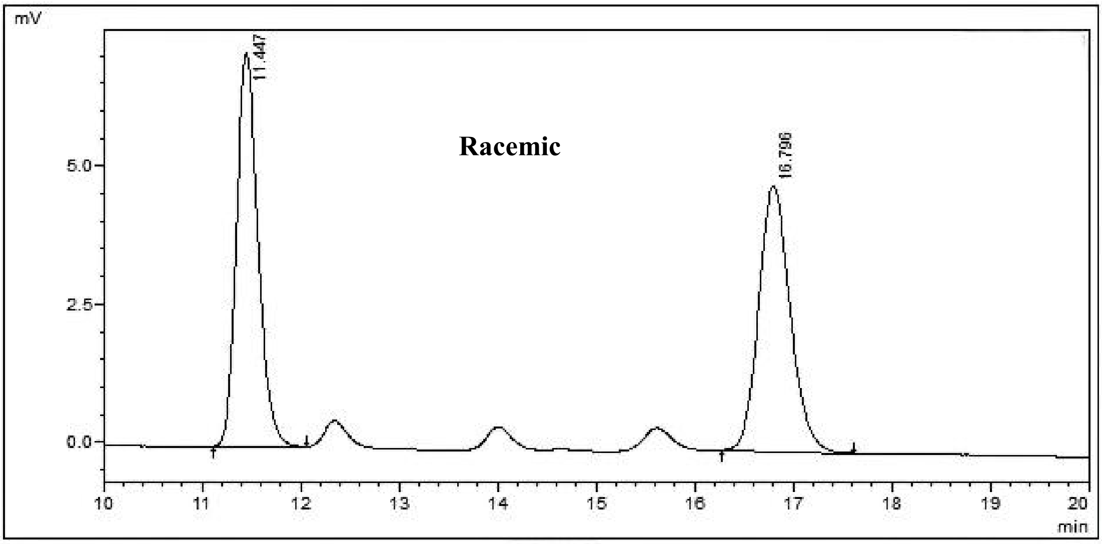

Ch1 238nm

	Ret. time (min)	Height (mv)	Width (min)	Area (mv*min)	Area%
1	11.447	7114	0.401	107597	50.242
2	16.796	4803	0.586	106561	49.758

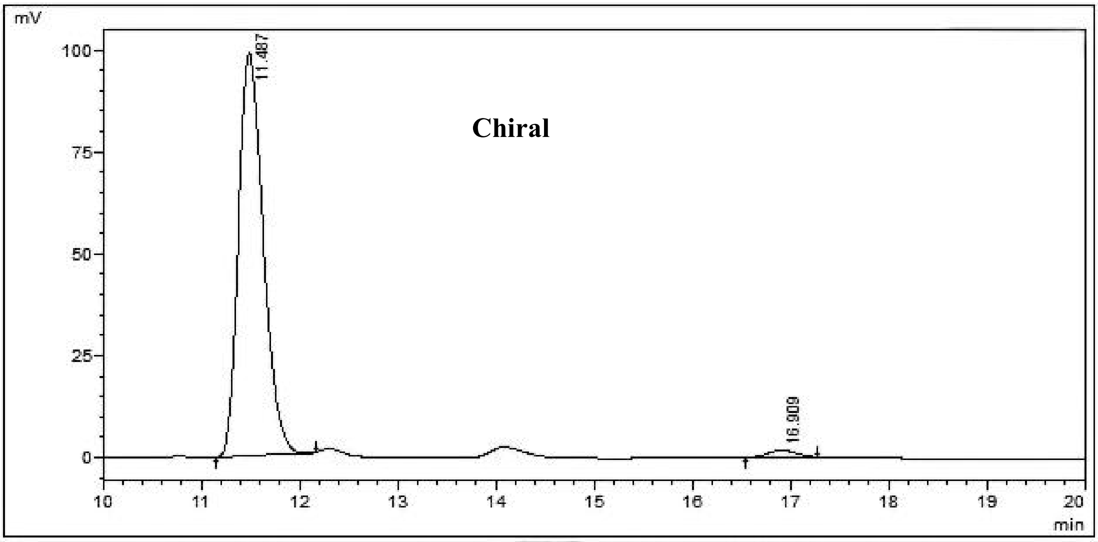

Ch1 238nm

	Rea. time (min)	Height (mv)	Width (min)	Area (mv*min)	Area %
1	11.487	98856	0.468	1731171	97.907
2	16.909	1760	0.576	37006	2.093

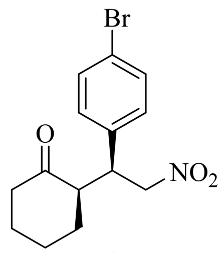

3g

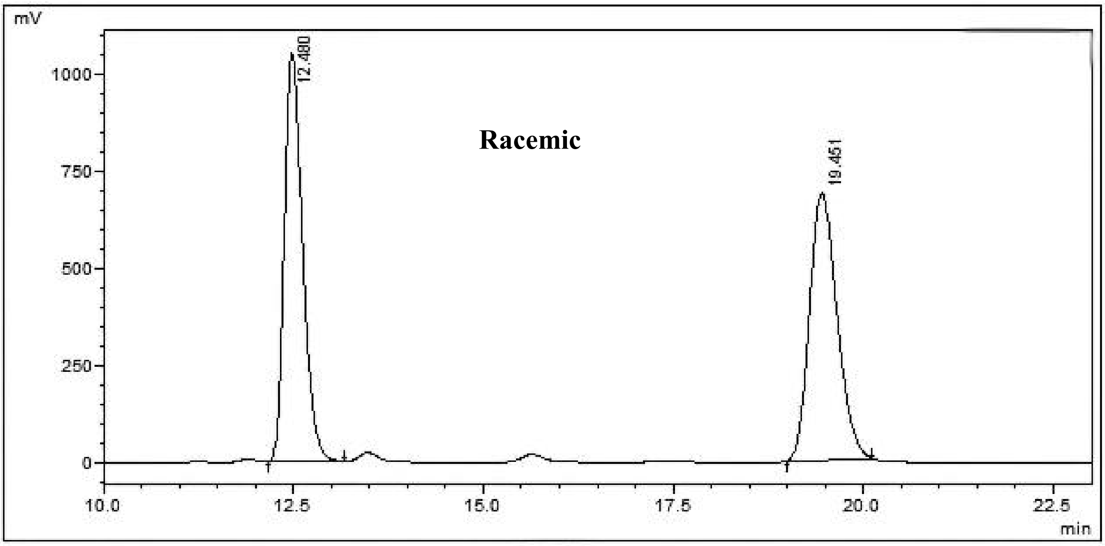

Ch1 220nm

	Ret. time (min)	Height (mv)	Width (mv)	Area (mv*min)	Area %
1	12.480	1051054	0.444	17609345	50.365
2	19.451	688155	0.670	17354309	49.635

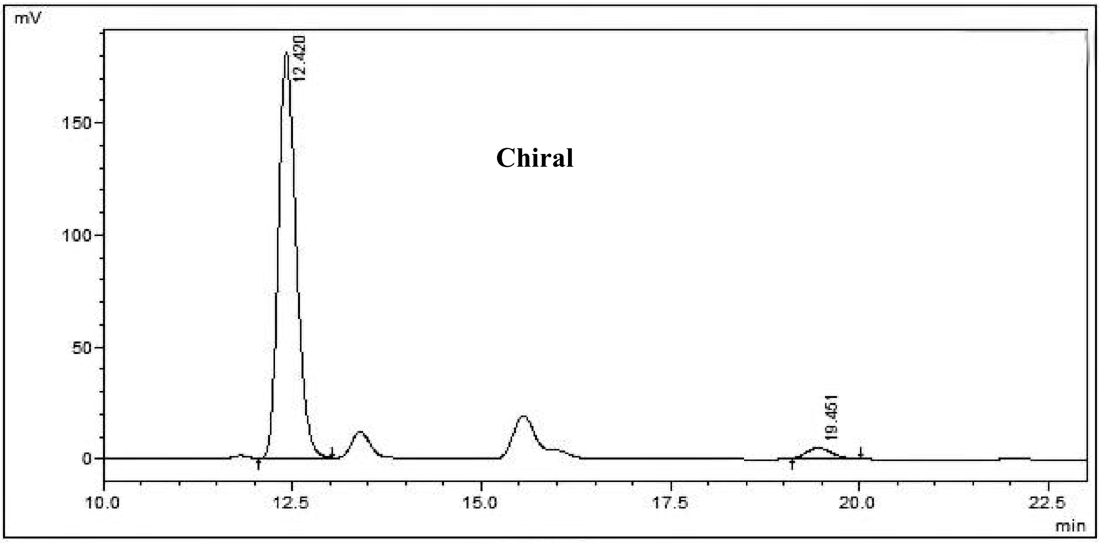

Ch1 220nm

	Ret. time (min)	Height (mv)	Width (mv)	Area (mv*min)	Area %
1	12.420	180727	0.430	2935317	96.215
2	19.451	4870	0.641	115462	3.785

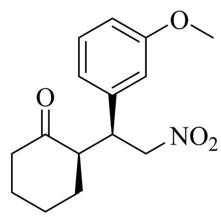

3h

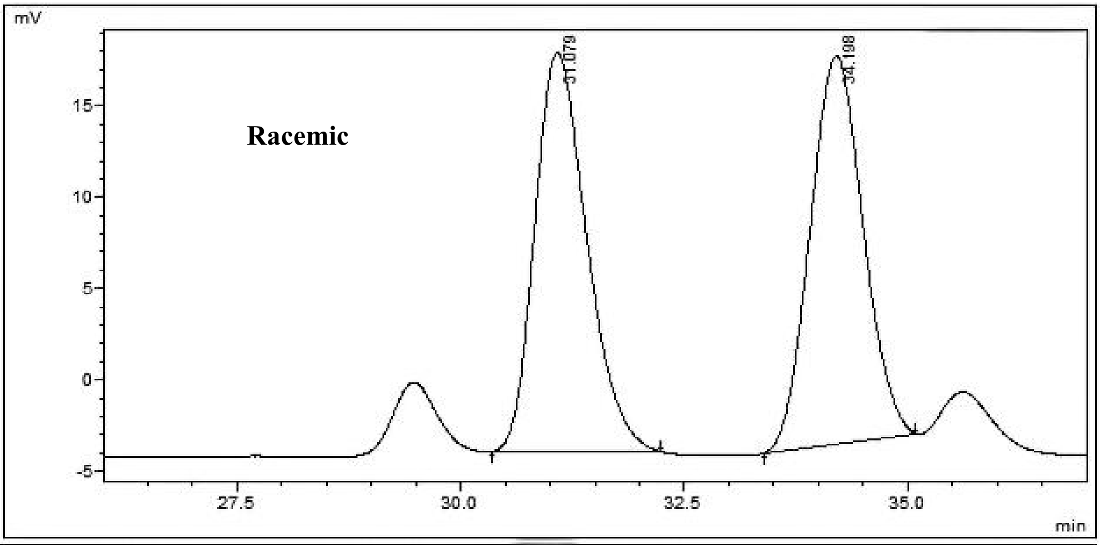

Ch1 254nm

	Ret. time (min)	Height (mv)	width (min)	Area (mv*min)	Area %
1	31.079	21817	1.070	884829	50.224
2	34.198	21237	1.109	876926	49.776

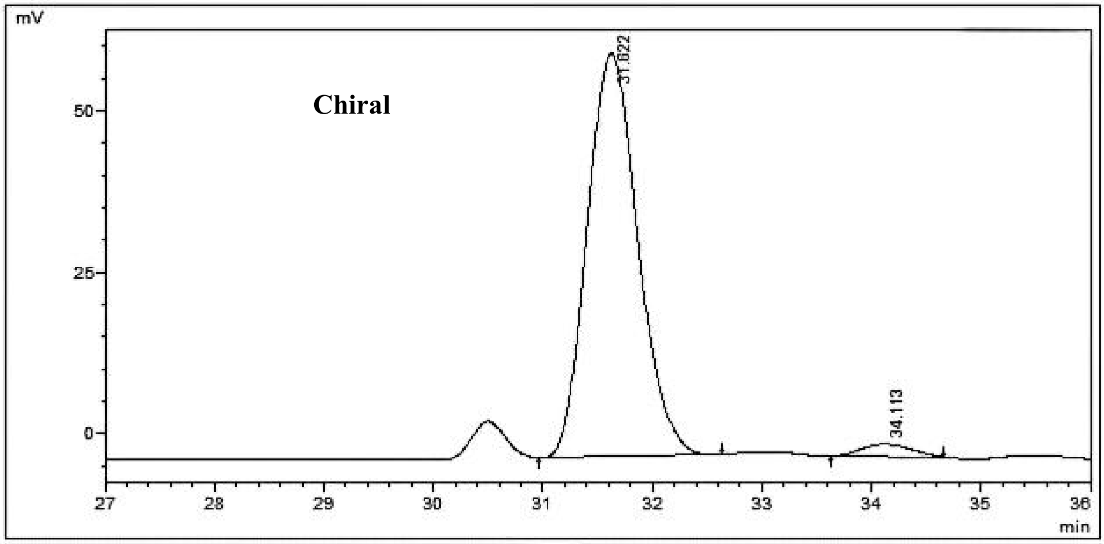

Ch1 254nm

	Ret. time (min)	Height (mv)	Width (min)	Area (mv*min)	Area %
1	31.622	62423	0.856	2009118	97.047
2	34.113	1928	0.874	61125	2.953

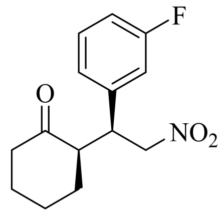

3i

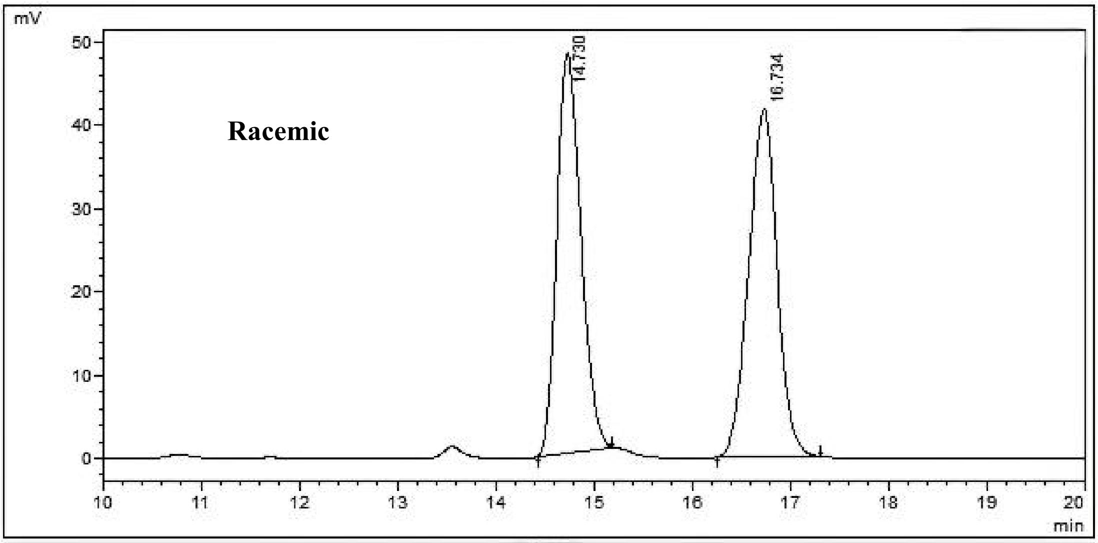

Ch1 254nm

	Ret. time (min)	Height (mv)	Width (min)	Area (mv*min)	Area %
1	14.730	47955	0.457	816123	49.069
2	16.734	41772	0.541	847100	50.931

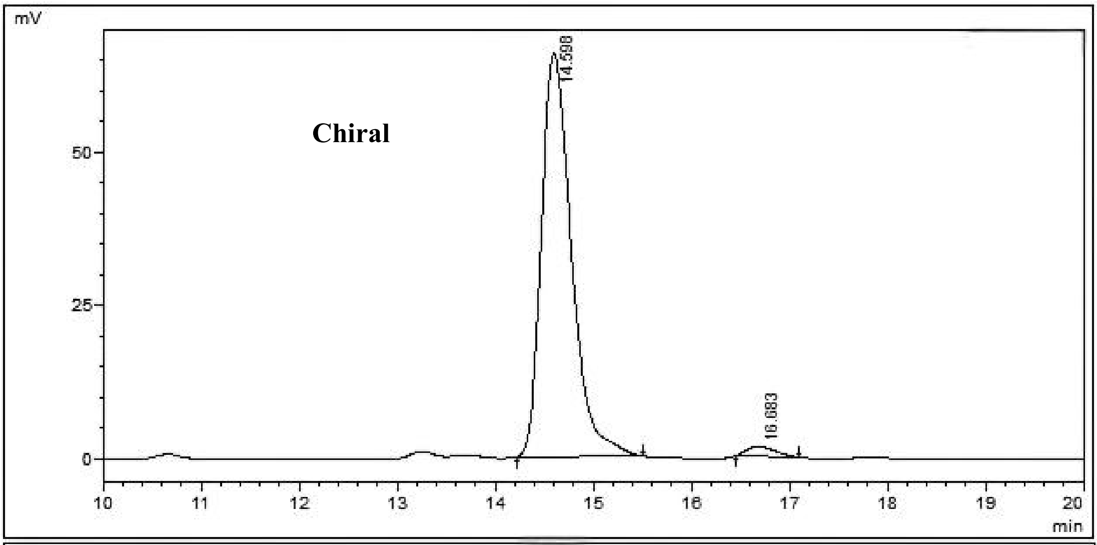

Ch1 254nm

	Ret. time (min)	Height (mv)	Width (min)	Area (mv*min)	Area %
1	14.598	65764	0.550	1403824	97.696
2	16.683	1630	0.565	33109	2.304

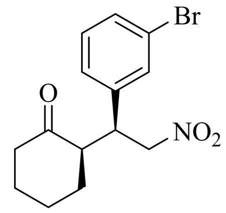

3j

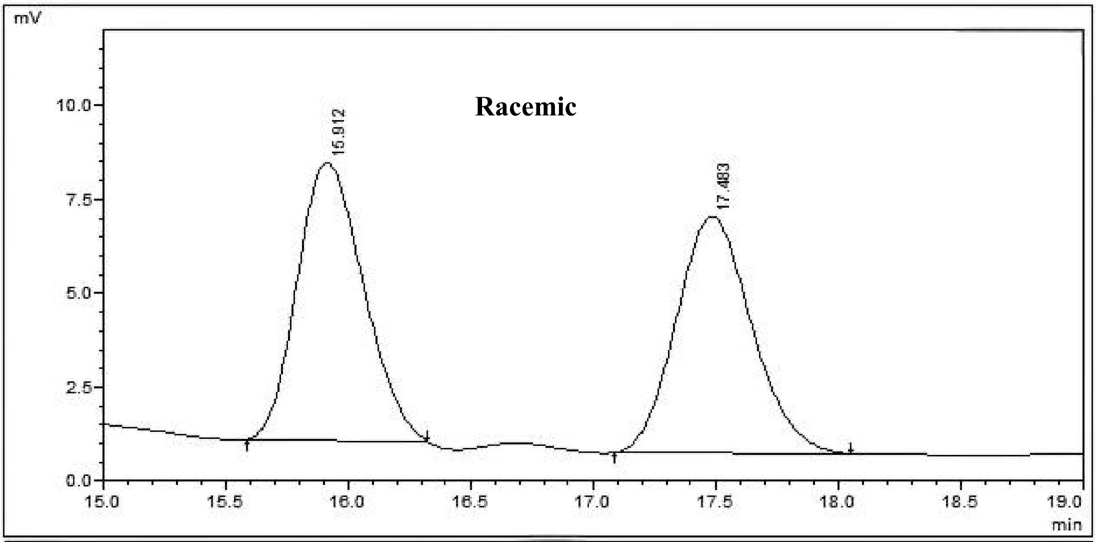

Ch1 254nm

	Ret. time (min)	Height (mv)	Width (min)	Area (mv*min)	Area %
1	15.912	7390	0.511	139163	50.799
2	17.483	6292	0.573	134785	49.201

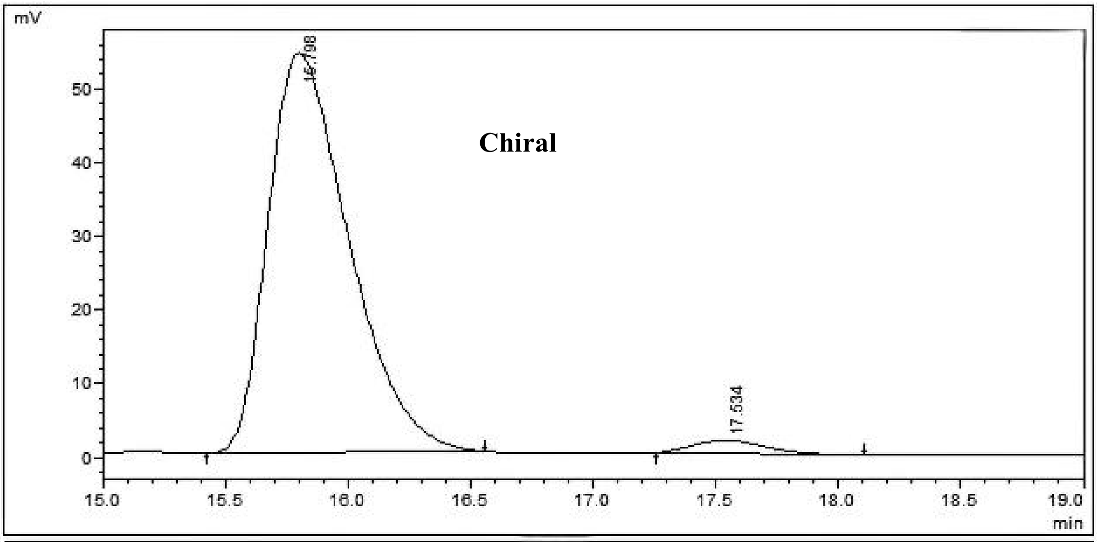

Ch1 254nm

	Ret. time (min)	Height (mv)	Width (min)	Area (mv*min)	Area %
1	15.798	54204	0.608	1242819	97.281
2	17.534	1751	0.544	34742	2.719

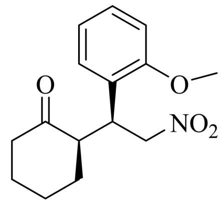

3k

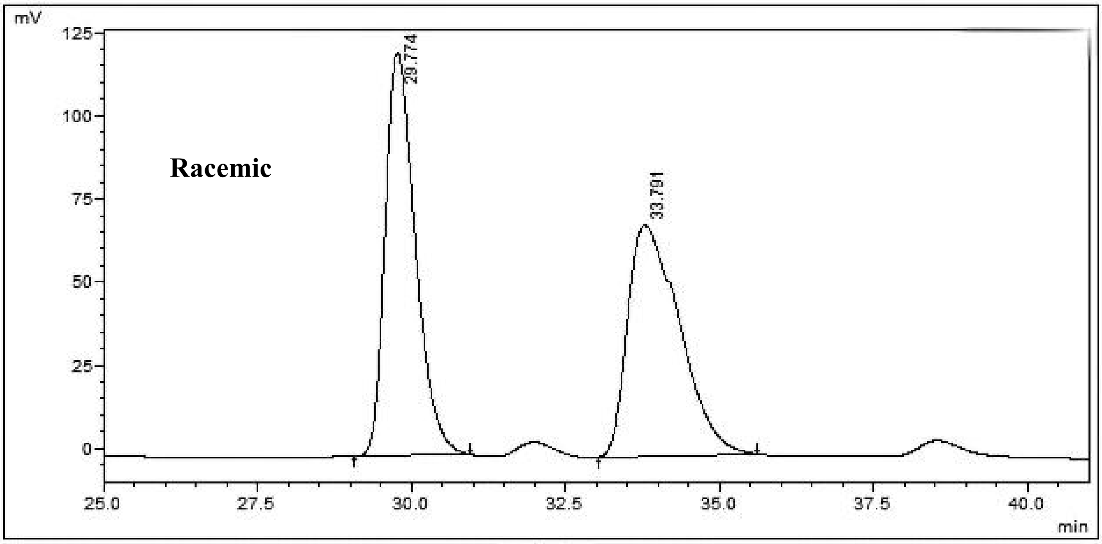

Ch1 220nm

	Ret. time (min)	Height (mv)	Width (min)	Area (mv*min)	Area %
1	29.774	120867	0.891	4121530	50.258
2	33.791	69459	1.528	4079144	49.742

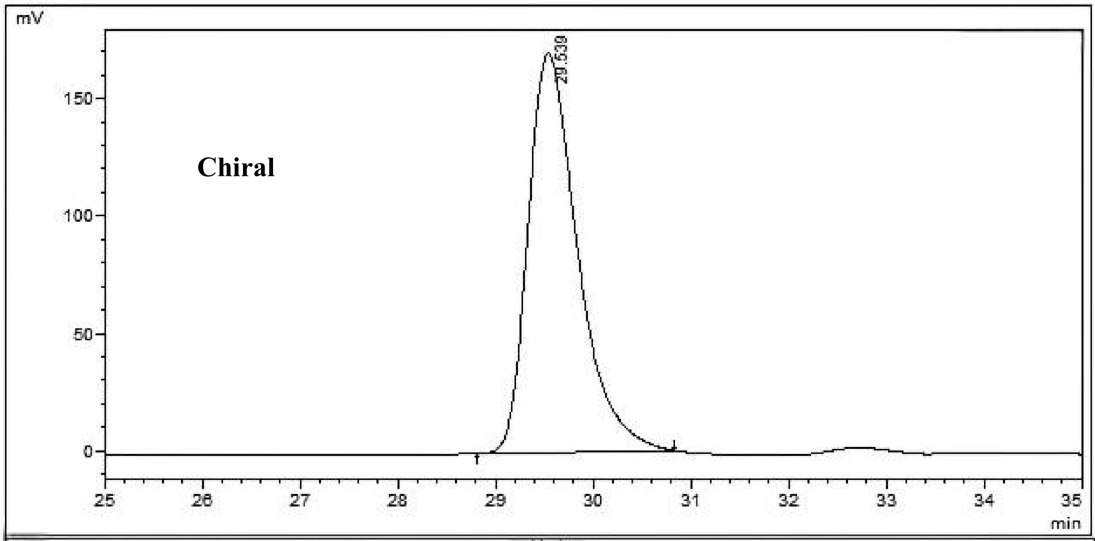

Ch1 220nm

	Ret. time (min)	Height (mv)	Width (min)	Area (mv*min)	Area %
1	29.539	169805	0.902	5992426	100.000

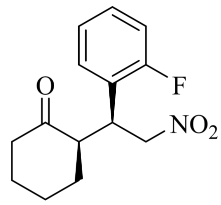

3l

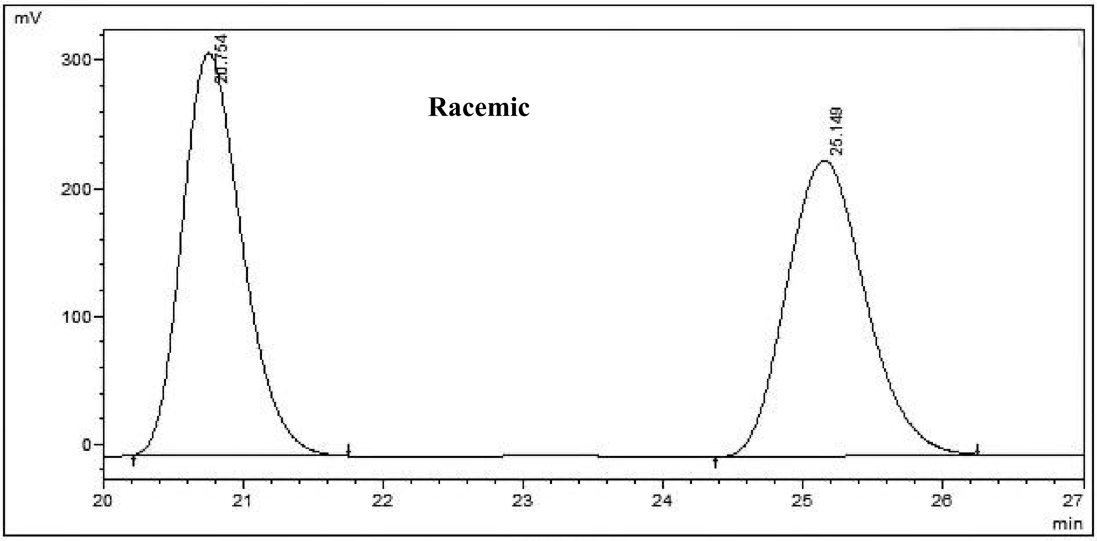

Ch1 220nm

	Ret. time (min)	Height (mv)	Width (min)	Area (mv*min)	Area %
1	20.754	314352	0.787	9364817	50.887
2	25.149	231206	1.030	9038247	49.113

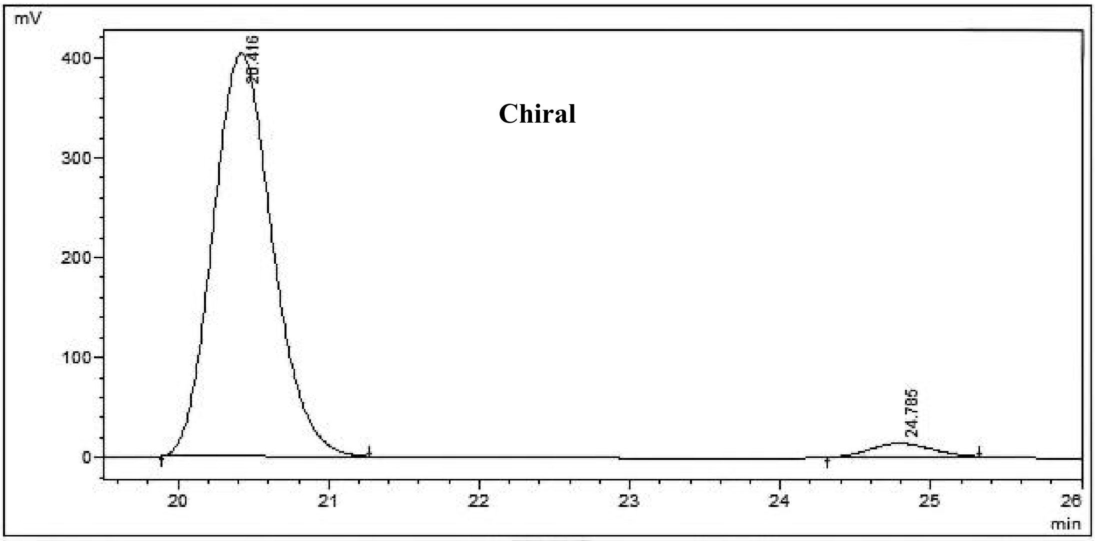

Ch1 220nm

	Ret. time (min)	Height (mv)	Width (min)	Area (mv*min)	Area %
1	20.416	402883	0.725	11058340	96.593
2	24.785	13584	0.784	390030	3.407

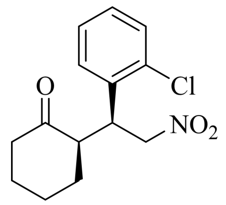

3m

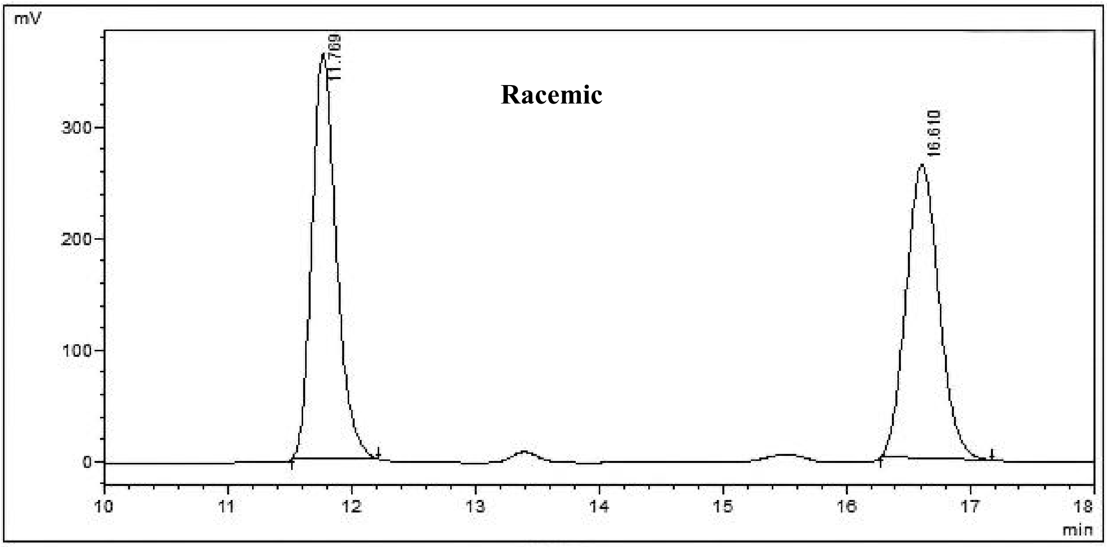

Ch1 220nm

	Ret. time (min)	Height (mv)	Width (min)	Area (mv*min)	Area %
1	11.769	363333	0.361	4997845	50.760
2	16.610	262804	0.492	4848281	49.240

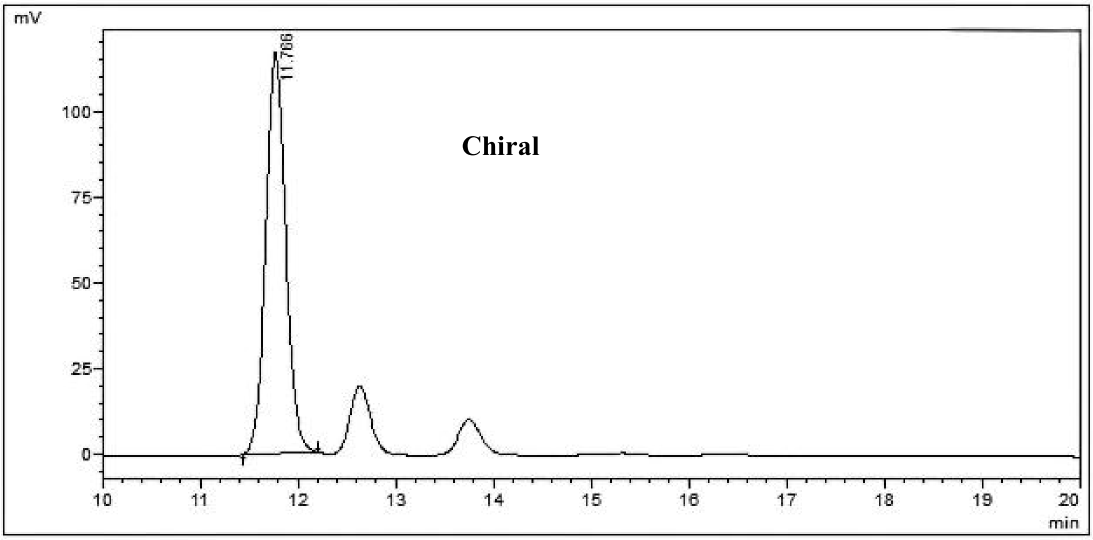

Ch1 220nm

	Ret. time (min)	Height (mv)	Width (min)	Area (mv*min)	Area %
1	11.766	116661	0.376	1651942	100.000

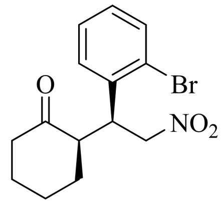

3n

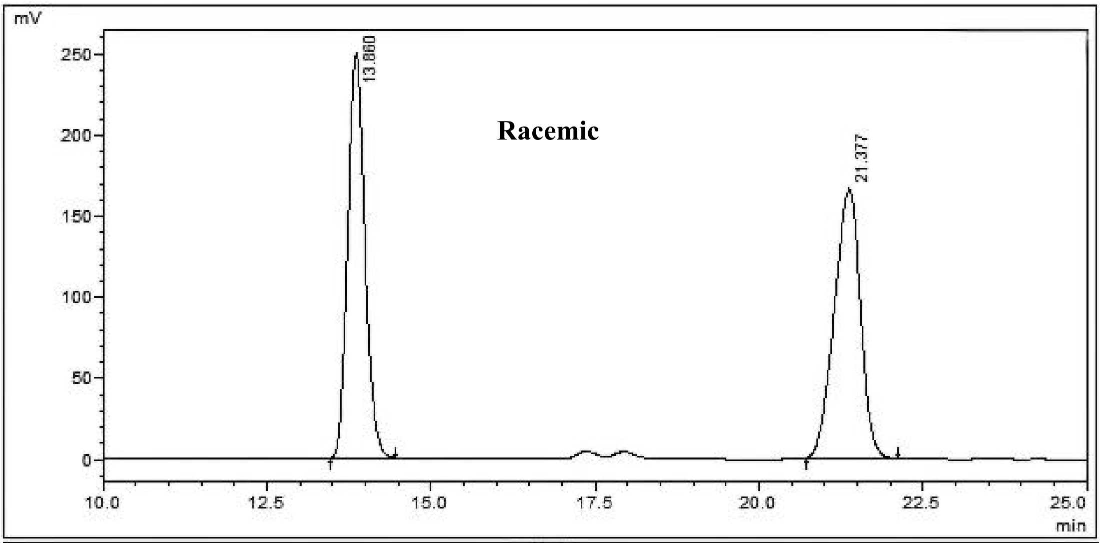

Ch1 238nm

	Ret. time (min)	Height (mv)	Width (min)	Area (mv*min)	Area %
1	13.860	249606	0.483	4545242	49.618
2	21.377	166148	0.748	4615284	50.382

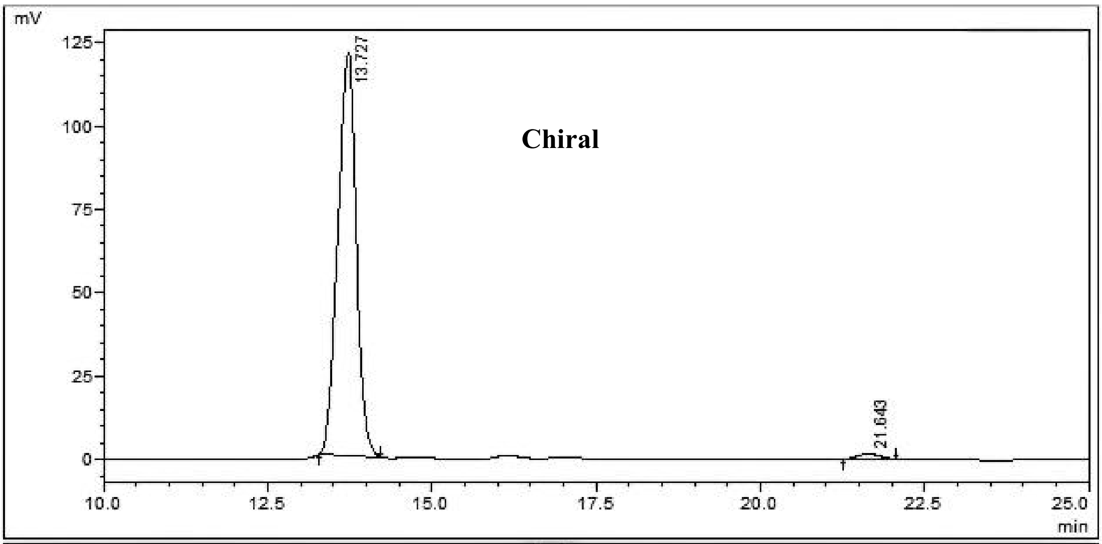

Ch1 254nm

	Ret. time (min)	Height (mv)	Width (min)	Area (mv*min)	Area %
1	13.727	120634	0.527	2347420	98.378
2	21.643	1559	0.684	38693	1.622

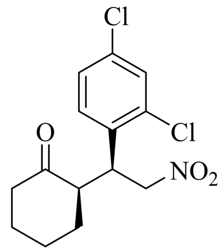

3o

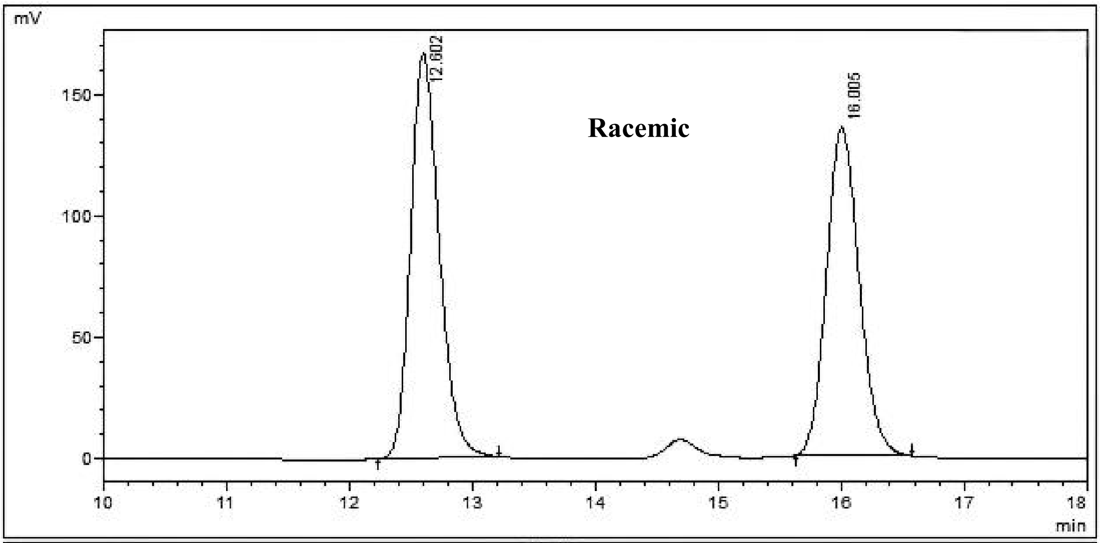

Ch1 220nm

	Ret. time (min)	Height (mv)	Width (min)	Area (mv*min)	Area %
1	12.602	166759	0.427	2699772	51.915
2	16.005	135223	0.492	2500617	48.085

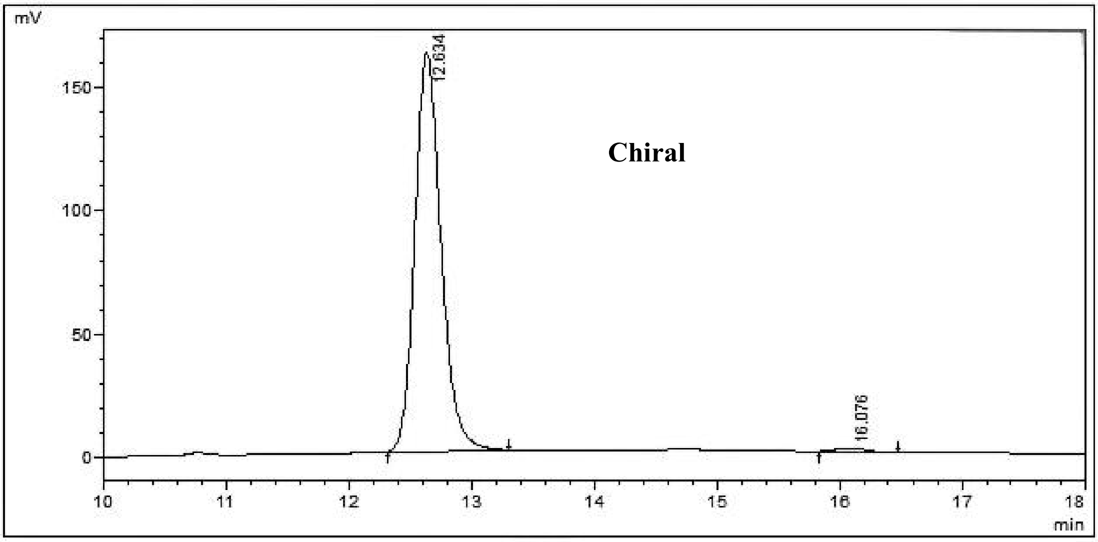

Ch1 220nm

	Ret. time (min)	Height (mv)	Width (min)	Area (mv*min)	Area %
1	12.634	160989	0.389	2386513	98.956
2	16.076	1551	0.447	25170	1.044

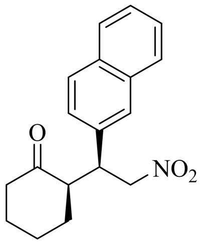

3p

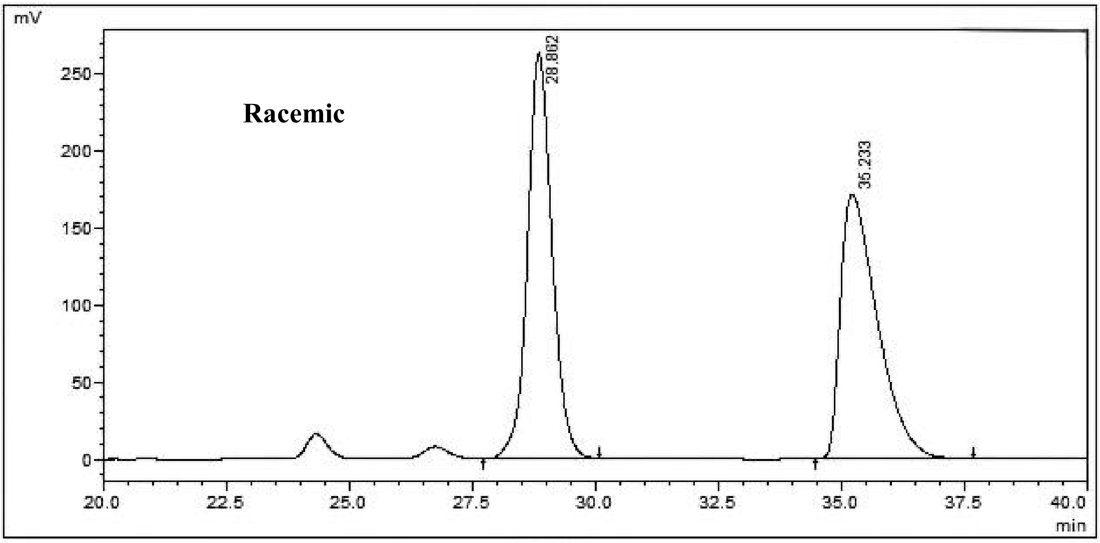

	Ch1 238nm Ret, time (min)	Height (mv)	Width (min)	Area (mv*min)	Area %
1	28.862	262945	0.892	9154491	50.448
2	35.233	171591	1.374	8991771	49.552

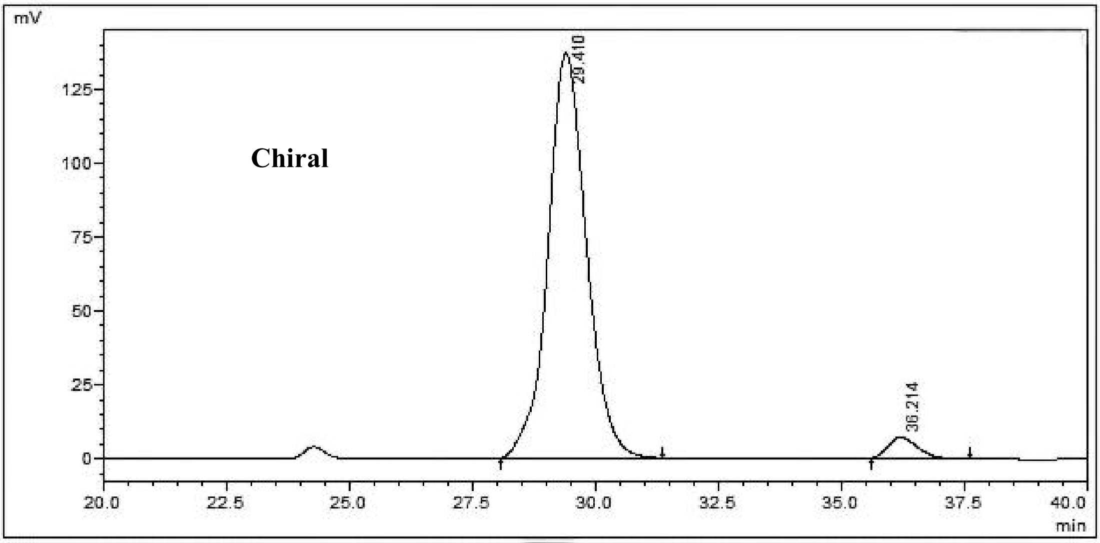

	Ch1 238nm Ret. time (min)	Height (mv)	Width (min)	Area (mv*min)	Area %
1	29.410	136929	1.384	7463906	96.241
2	36.214	7085	1.099	291505	3.759

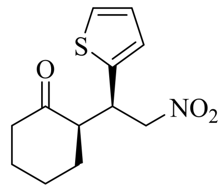

3q

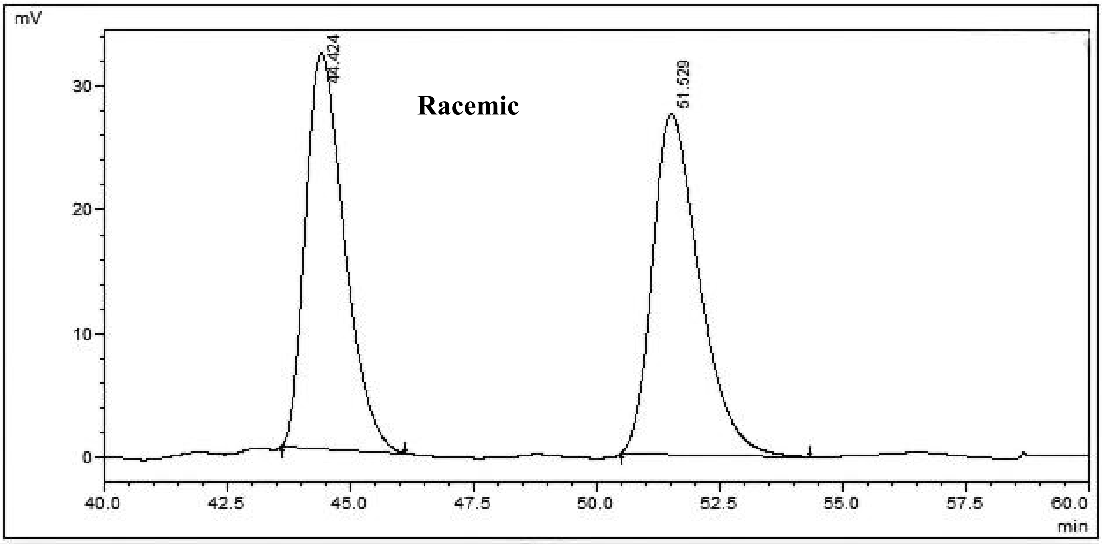

Ch1 254nm

	Ret. time (min)	Height (mv)	Width (min)	Area (mv*min)	Area %
1	44.424	31923	1.459	1764083	49.159
2	51.529	27508	1.709	1824458	50.841

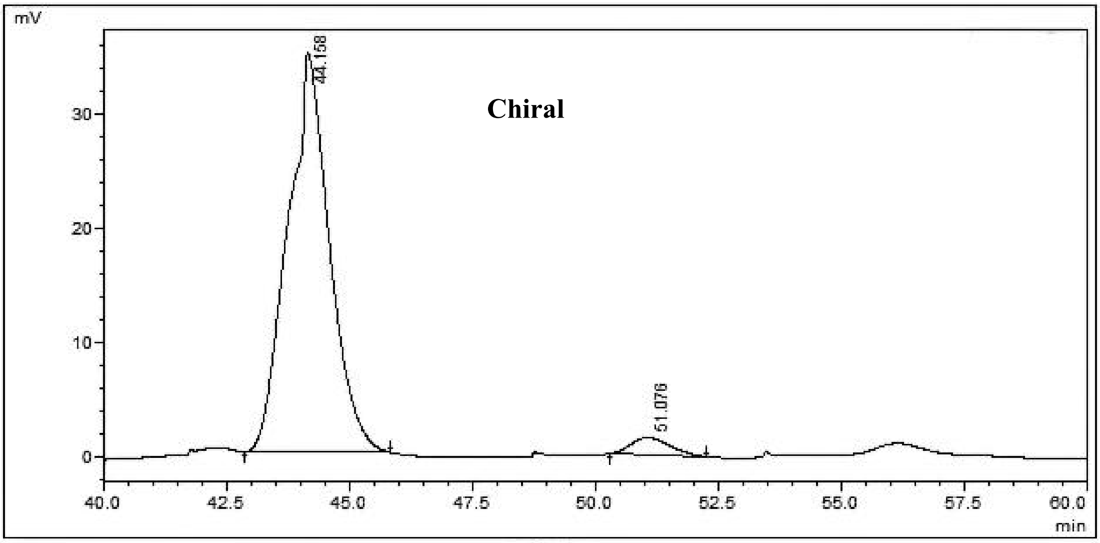

Ch1 254nm

	Ret. time (min)	Height (mv)	Width (min)	Area (mv*min)	Area %
1	44.158	34799	1.736	2046168	96.073
2	51.076	1512	1.510	83638	3.927

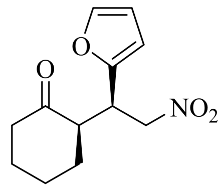

3r

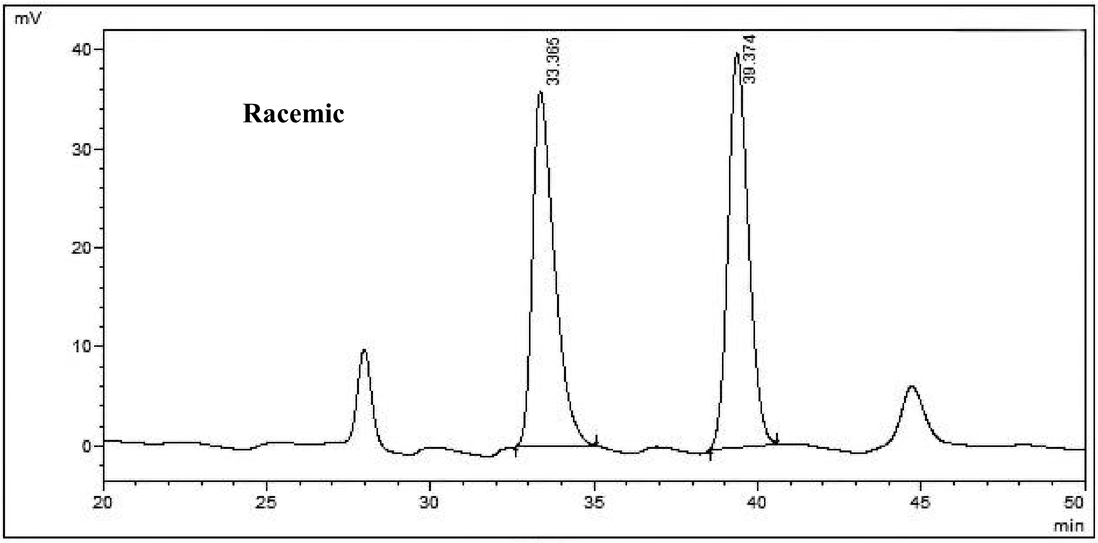

Ch1 220nm

	Ret. time (min)	Height (mv)	Width (min)	Area (mv*min)	Area%
1	33.365	35813	1.217	1664743	49.133
2	39.374	39774	1.145	1723462	50.867

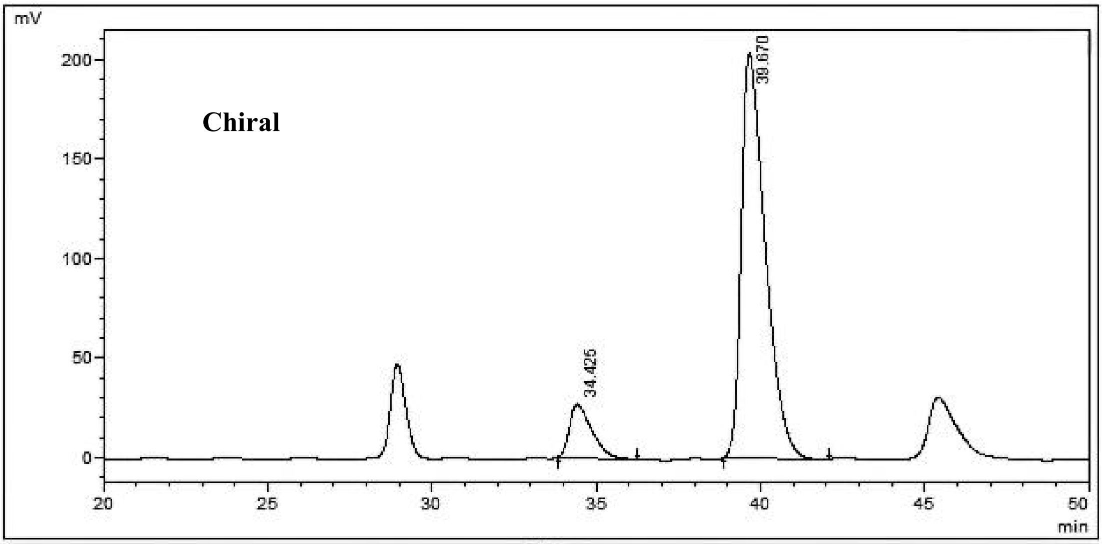

Ch1 220nm

	Ret. time (min)	Height (min)	Width (min)	Area (mv*min)	Area %
1	34.425	27169	1.243	1282572	11.085
2	39.670	203399	1.326	10287556	88.915

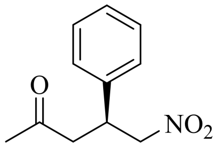

4

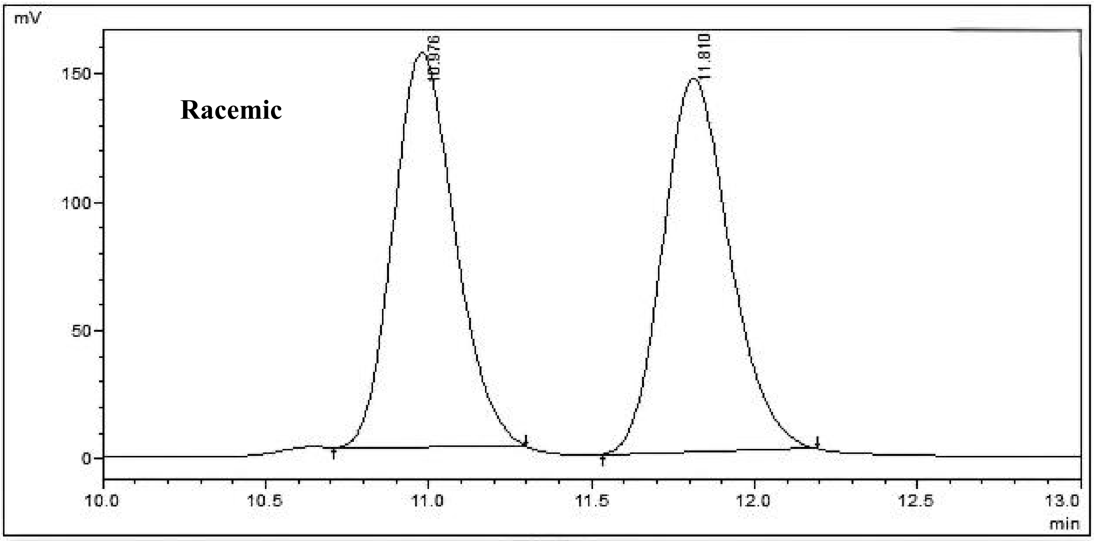

Ch1 254nm

	Ret. time (min)	Height (mv)	Width (min)	Area (mv*min)	Area%
1	10.976	153310	0.357	2026157	49.251
2	11.810	145672	0.384	2087748	50.749

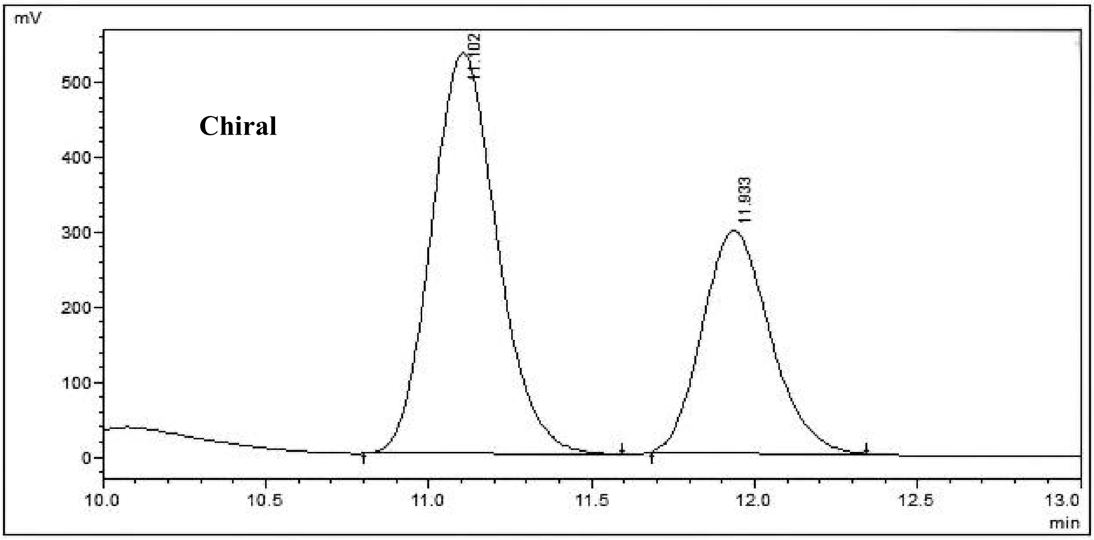

Ch1 254nm

	Ret. time (min)	Height (mv)	Width (min)	Area (mv*min)	Area%
1	11.102	534602	0.375	7524559	63.674
2	11.933	296782	0.388	4292824	36.326
